# Targeting Mammalian 5-Lipoxygenase by Dietary Phenolics as an Anti-Inflammatory Mechanism: A Systematic Review

**DOI:** 10.3390/ijms22157937

**Published:** 2021-07-25

**Authors:** Juan Antonio Giménez-Bastida, Antonio González-Sarrías, José Moisés Laparra-Llopis, Claus Schneider, Juan Carlos Espín

**Affiliations:** 1 Laboratory of Food and Health, Research Group on Quality, Safety and Bioactivity of Plant Foods, Department Food Science and Technology, CEBAS-CSIC, P.O. Box 164, Campus de Espinardo, 30100 Murcia, Spain; jcespin@cebas.csic.es; 2 Group of Molecular Immunonutrition in Cancer, Madrid Institute for Advanced Studies in Food (IMDEA-Food), 28049 Madrid, Spain; moises.laparra@imdea.org; 3 Division of Clinical Pharmacology, Department of Pharmacology, Vanderbilt Institute of Chemical Biology, Vanderbilt University Medical School, Nashville, TN 37232, USA; claus.schneider@vanderbilt.edu

**Keywords:** 5-LOX, polyphenols, inflammation, leukotrienes, eicosanoids, hemiketals, arachidonic acid

## Abstract

5-Lipoxygenase (5-LOX) plays a key role in inflammation through the biosynthesis of leukotrienes and other lipid mediators. Current evidence suggests that dietary (poly)phenols exert a beneficial impact on human health through anti-inflammatory activities. Their mechanisms of action have mostly been associated with the modulation of pro-inflammatory cytokines (TNF-α, IL-1β), prostaglandins (PGE_2_), and the interaction with NF-κB and cyclooxygenase 2 (COX-2) pathways. Much less is known about the 5-lipoxygenase (5-LOX) pathway as a target of dietary (poly)phenols. This systematic review aimed to summarize how dietary (poly)phenols target the 5-LOX pathway in preclinical and human studies. The number of studies identified is low (5, 24, and 127 human, animal, and cellular studies, respectively) compared to the thousands of studies focusing on the COX-2 pathway. Some (poly)phenolics such as caffeic acid, hydroxytyrosol, resveratrol, curcumin, nordihydroguaiaretic acid (NDGA), and quercetin have been reported to reduce the formation of 5-LOX eicosanoids in vitro. However, the in vivo evidence is inconclusive because of the low number of studies and the difficulty of attributing effects to (poly)phenols. Therefore, increasing the number of studies targeting the 5-LOX pathway would largely expand our knowledge on the anti-inflammatory mechanisms of (poly)phenols.

## 1. Introduction

### 1.1. Lipoxygenases

Lipoxygenases (LOXs) are found widely in nature and are abundant in plants and animals. Polyunsaturated fatty acids (PUFA) containing *cis* double bonds are the substrates of these enzymes. Linoleic and linolenic acids (18-carbon fatty acids) and arachidonic acid (AA; 20-carbon fatty acid) are the most common substrates for LOXs in plants and animals, respectively. The nomenclature of these enzymes is based on the specific position of the carbon oxygenated. Some examples are 9-LOX and 13-LOX, which are important LOXs described in plants, whereas 5-LOX, 12-LOX, and 15-LOX are present in animals [1,2]. 

LOXs are involved in the modulation of essential biological functions by synthesizing specific hydroperoxides, which are further metabolized into signaling molecules/biological mediators. Among these molecules, divinyl ethers, aldehydes, and jasmonates exert protective effects in plants from abiotic stress and(or) pathogens [3,4], whereas lipoxins or leukotrienes modulate the inflammatory response in humans [5]. LOX-catalyzed reactions are also associated with undesirable effects. Legume spoilage, generation of hay-like flavor, loss of pigments (e.g., carotenoids and chlorophylls), enzymatic browning and/or rancidity are effects linked to LOX oxidation (together with other oxidases) in plants [2]. In humans, an exacerbated activation of 5-LOX produces elevated levels of leukotrienes (LTs) promoting inflammation and related diseases (e.g., bronchoconstriction) [6,7]. 

The inhibition of LOX-mediated oxidation is an interesting strategy to minimize/avoid the loss of quality of plant-derived foodstuff. Current techniques for inhibition of LOX oxidation include the assay of phenolic compounds, which exert their protective effects through their antioxidant activity [2]. The structural similarities and mechanisms of action between plant and animals LOXs [8] suggest that the phenolic compounds might interfere with animal LOXs, including 5-LOX. However, the mechanisms by which phenolic compounds modulate 5-LOX (and the inflammatory response) go beyond their antioxidant activity, as described in this review.

### 1.2. 5-Lipoxygenase and Inflammation

Inflammation is a complex physiological process that functions as a network of interconnected elements regulated by many signaling molecules, including cytokines, chemokines, and lipid mediators. The disruption of the equilibrium between these molecules results in chronic inflammation and the development of related diseases [9,10]. AA is a substrate for the biosynthesis of several groups of lipid mediators collectively termed eicosanoids. The formation of prostaglandins (PGs) and LTs are two major pathways of eicosanoid biosynthesis catalyzed by cyclooxygenases (COX)-1/COX-2 and 5-lipoxygenase (5-LOX), respectively. The established role of the latter enzyme is its contribution to (patho)-physiological inflammation by the formation of LTs [11,12]. The enzyme 5-LOX is also central for the biosynthesis of the more recently discovered (and less investigated) 5-LOX-derived metabolites termed hemiketal (HK) eicosanoids [13] that appear to be novel lipid mediators in inflammation. LTs, at low nanomolar concentrations, can modulate the immune response and promote chronic inflammation, implying a role of these eicosanoids in a range of inflammatory diseases, including atherosclerosis, inflammatory bowel diseases, rheumatoid arthritis, and asthma [14,15]. The understanding of the biology of 5-LOX and its LT products has culminated in the development of anti-LT drugs (receptor antagonist and enzyme inhibitors) that are used clinically in the treatment of asthma and that may also provide a clinical benefit in atherosclerosis [11,16,17]. However, although these drugs show therapeutic effects (e.g., against asthma and atherosclerosis), the side-effects associated with their use and/or the poor in vivo efficacy highlight the need for better therapeutic options, including the search for possible alternatives such as natural products that may include dietary (poly)phenolic compounds.

In this regard, dietary (poly)phenols have been tested in numerous preclinical (animal and cellular) models and in a limited number of human studies, showing anti-inflammatory effects by diverse mechanisms of action, including cytokine modulation, inhibition of inducible nitric oxide synthase (iNOS) and nuclear factor kappa B (NF-κB) activation, as well as decreasing PG production by down-regulation of COX-2 [18,19]. Much less is known about the anti-inflammatory effect of (poly)phenols targeting the 5-LOX pathway. It is noteworthy that the number of studies that have investigated the anti-inflammatory effects of dietary (poly)phenols on 5-LOX (around 120 hits for a PubMed search) is much lower compared to COX-2 (around 2500 studies). The preponderance of a focus on the COX-2 pathway is difficult to rationalize given the importance of targeting both COX-2 and 5-LOX pathways to ameliorate undesirable effects of chronic inflammation.

Accordingly, our main objective was to perform a systematic and critical review of the current evidence concerning the anti-inflammatory effect of dietary (poly)phenolics via modulation of the 5-LOX pathway to identify knowledge gaps and future research needs, allowing an increase in the understanding of the anti-inflammatory effects of (poly)phenols.

### 1.3. 5-Lipoxygenase Pathway

#### 1.3.1. Biosynthesis of 5-LOX-Derived Eicosanoids

Early studies on LT biosynthesis date back to the 1970s when Samuelsson and Borgeat described the formation of 5*S*-hydroxyeicosatetraenoic acid (5S-HETE) together with new arachidonate metabolites that were later termed LTA_4_ and LTB_4_ in peripheral leukocytes [20,21]. These investigations established a novel pathway of oxidative transformation of AA catalyzed by 5-LOX.

**Figure 1:**
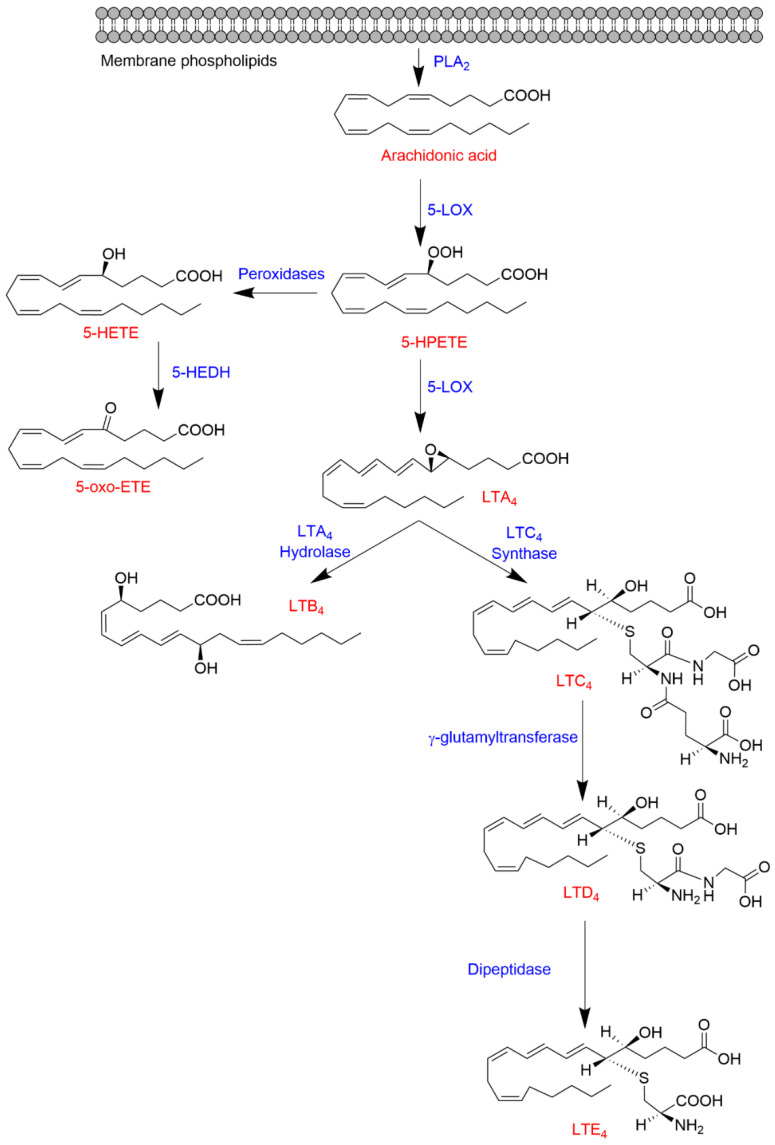
Biosynthesis of 5-HETE, 5-oxo-HETE, and LTs from arachidonic acid.

5-LOX is a key enzyme in the biosynthesis of LTs from AA (Figure 1). The formation of LTs requires activation of phospholipase A_2_ (PLA_2_) by Ca^+2^-dependent (such as purinergic stimulation by ATP) or independent (i.e., innate immune “toll-like” receptor (TLR) stimulation by LPS) mechanisms, resulting in the hydrolysis of AA esterified in the membrane phospholipids [22,23]. In intact cells, 5-LOX is activated in response to Ca^+2^ influx and associates with 5-lipoxygenase activating protein (FLAP) to form a 5-LOX/FLAP complex at the nuclear membrane. In this complex, the essential function of FLAP is to present AA as a substrate to 5-LOX. The 5-LOX catalytic reaction involves an initial hydrogen abstraction from carbon 7 of AA and the addition of molecular oxygen to produce 5*S*-hydroperoxyeicosatetraenoic acid (5*S*-HPETE) followed by a second hydrogen abstraction from position 10 to form LTA_4_. LTA_4_ is unstable and undergoes enzymatic transformation by LTA_4_ hydrolase to form LTB_4_ or conjugation with glutathione by LTC_4_ synthase to produce LTC_4_, which is further metabolized by γ-glutamyltransferase and dipeptidase yielding LTD_4_ and LTE_4_, respectively [24,25]. Alternatively, 5*S*-HPETE can undergo reduction to 5S-HETE, which is in turn oxidized by 5-hydroxyeicosanoid dehydrogenase (5-HEDH), yielding 5-oxo-eicosatetraenoic acid (5-oxo-ETE) [26] (Figure 1).

#### 1.3.2. Transcellular Biosynthesis of Leukotrienes and Lipoxins

LT biosynthesis goes beyond a string of enzymatic transformations in single cells (i.e., granulocytes and mast cells). Namely, LT biosynthesis involves transcellular biosynthesis, a term that describes eicosanoid formation by cell–cell interactions [27]. Early evidence on transcellular biosynthesis of LTs came from in vitro studies describing LTA_4_ exchange between neutrophils and erythrocytes or endothelial cells to produce LTB_4_ or LTC_4_, respectively [28,29]. Subsequent in vivo studies provided evidence that transcellular biosynthesis of LTB_4_ and LTC_4_ does indeed occur in animal models [30,31].

**Figure 2:**
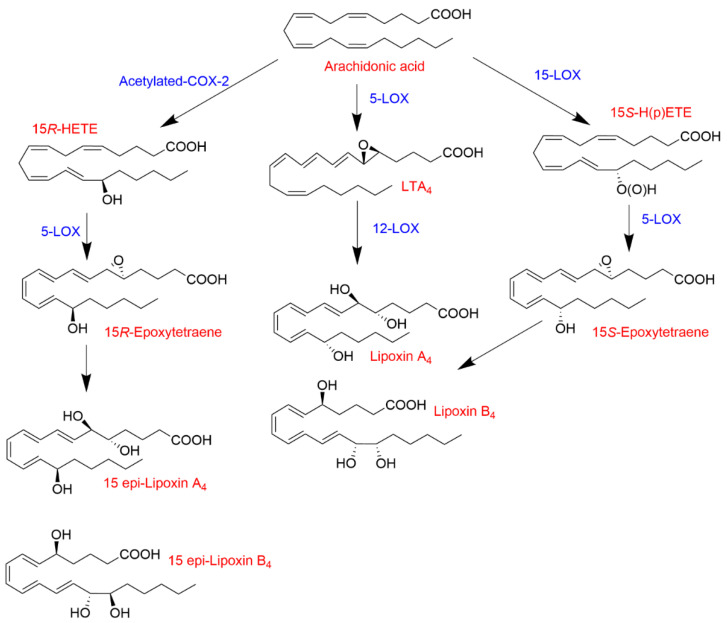
Biosynthesis of LXs and aspirin-triggered LXs.

The formation of lipoxins (lipoxygenase interaction products; LXs) is another paradigmatic example of transcellular biosynthesis (Figure 2). Synthesis of LXs requires the combination of different cell types (i.e., neutrophils, endothelial cells, and(or) platelets) expressing different lipoxygenases (i.e., 5-LOX, 12-LOX and(or) 15-LOX) [32]. For example, the tandem interaction of 5-LOX- and 12-LOX-expressing cells (i.e., neutrophils and platelets, respectively) leads to the formation of LXA_4_ from LTA_4_ [33,34]. Another transcellular mechanism of LX biosynthesis involves the interaction of 15-LOX (monocytes/macrophages and epithelial cells) and 5-LOX, resulting in the sequential transformation of AA to 15*S*-HPETE followed by the conversion by 5-LOX to yield LXA_4_ and LXB_4_ [35]. The synthesis of LXs is not limited to the interaction of only lipoxygenases. Acetylated COX-2 retains catalytic activity, forming 15*R*-HETE as the primary product [36,37], which can serve as a substrate for 5-LOX producing the aspirin-triggered LXs (LXA_4_ and LXB_4_ epimers) known as 15-epi-LXA_4_ and 15-epi-LXB_4_ [22,38,39]. LXs and aspirin-triggered LXs have anti-inflammatory properties promoting the resolution of inflammation [40,41,42], which contrasts with the largely pro-inflammatory effects of other products of the 5-LOX pathway.

#### 1.3.3. The 5-LOX/COX-2 Crossover Biosynthetic Pathway

**Figure 3:**
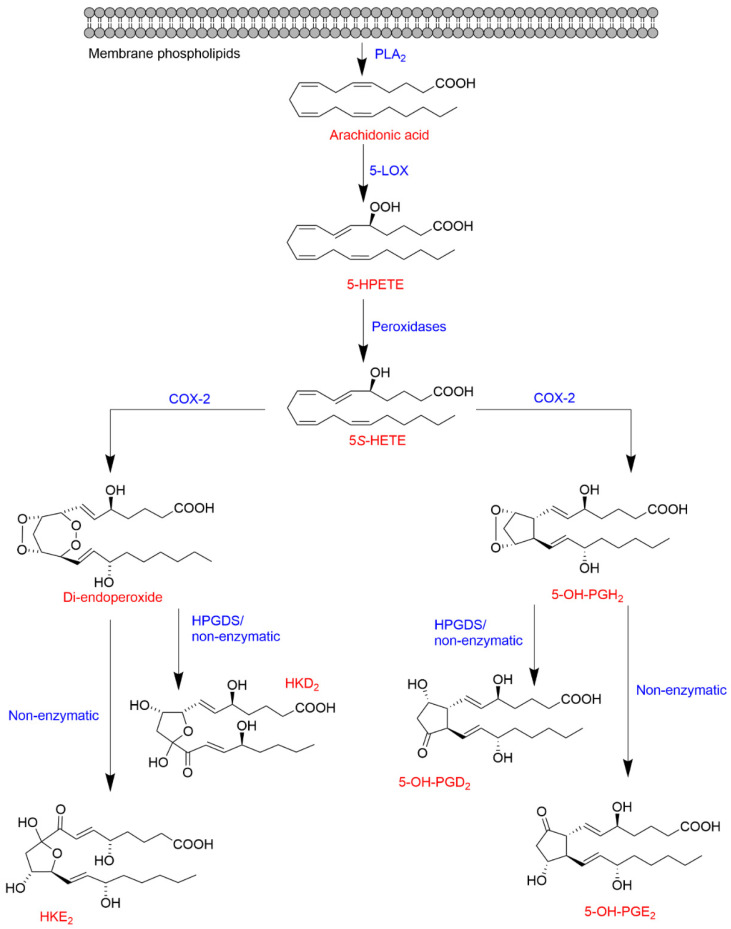
Biosynthetic crossover of the 5-LOX and COX-2 pathways yielding hemiketal eicosanoids and 5-hydroxy-prostaglandins.

Besides a role in the biosynthesis of LTs and LXs, 5-LOX is also a key enzyme in the biosynthesis of a novel type of eicosanoids recently described. In vitro biochemical studies showed that COX-2 catalyzes the oxidation of the 5-LOX product 5S-HETE (resulting from the reduction of 5S-HPETE) to form a di-endoperoxide [43] and 5-OH-PGH_2_ [44], which are equivalent to the prostaglandin endoperoxide PGH_2_ of the COX-2 pathway. The reaction also yields two minor compounds identified as 5,11- and 5,15-di-HETE, the 5-hydroxy-analogs of the known COX by-products, 11- and 15-HETE [45,46]. Additional studies of the 5-LOX/COX-2 crossover pathways described the in vitro transformation (enzymatic and non-enzymatic) of the di-endoperoxide to two hemiketals (HKs) named HKE_2_ and HKD_2_ [13] and of 5-OH-PGH_2_ to 5-OH-PGE_2_ and 5-OH-PGD_2_ [44] as shown in Figure 3.

**Figure 4:**
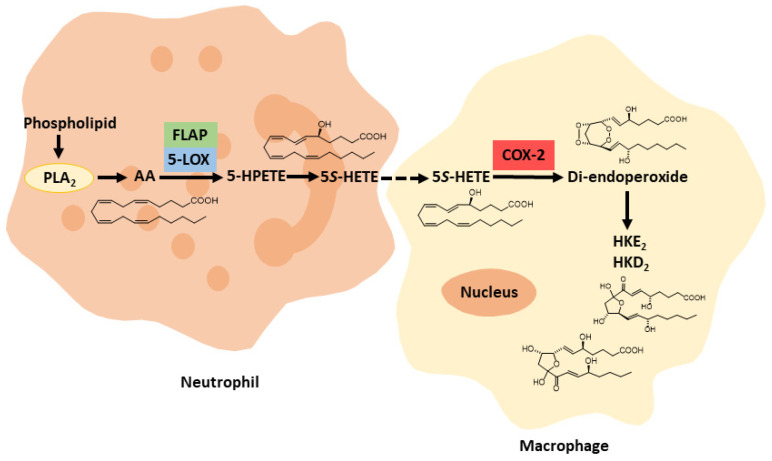
Proposed transcellular biosynthesis of hemiketal eicosanoids.

Biosynthesis of HKE_2_ and HKD_2_ was established using an ex vivo model of human isolated leukocytes, stimulated with calcium ionophore A23187 and LPS for 5-LOX activation and COX-2 up-regulation, respectively [13]. Analysis of the time-course of the formation of HKs in human leukocyte mixtures revealed that their biosynthesis mainly depends on the availability of the 5S-HETE substrate and, to a lesser extent, on the activity of COX-2 [47]. HK formation in the mixture of human leukocytes may be another example of transcellular biosynthesis, given that a single type of leukocyte is unlikely to exhibit a significant activity of both 5-LOX and COX-2. The dependence of HK biosynthesis on the activation of leukocyte mixtures by both A23187 (inducing 5-LOX) and LPS (inducing COX-2) suggests a role for neutrophils and activated monocytes/macrophages, respectively, although this was not directly established (Figure 4).

#### 1.3.4. Role of 5-Lipoxygenase-Derived Eicosanoids in Inflammation

Decades of intense investigation have identified LTs as potent inducers of inflammation through the interaction with distinct G protein-coupled receptors. LTB_4_ was one of the first chemotactic molecules identified [48] and is a well-known pro-inflammatory molecule that exerts its effects through interaction with its high-affinity receptor BLT1 [49,50]. LTB_4_ also binds (and activates) a second receptor, BLT2, albeit with much less affinity than that reported for BLT1, and its function via interaction with BLT2 remains elusive [51]. Thus, LTB_4_ promotes pro-inflammatory responses such as leukocyte chemoattraction, leukocyte–endothelial cell interaction, and the release of inflammatory mediators at inflammation sites [52]. Here, aberrant inflammation results in tissue damage and impairs adequate function of host innate immune effectors such as neutrophils and activated monocytes/macrophages to recognize, respond, and resolve inflammatory processes properly. This dysregulated response is implicated in the pathogenesis of chronic diseases such as atherosclerosis, cardiovascular, and inflammatory bowel diseases [15,53,54,55]. On the other hand, the cysteinyl-LTs (cysLTs), LTC_4_, LTD_4_, and LTE_4_ activate their cognate receptors, CysLT1 and CysLT2, and exert profound effects on airway inflammation leading to bronchoconstriction, vascular permeability, and neutrophil extravasation [6,15,56,57,58,59].

5-HETE is a particular eicosanoid that shows limited biological activity by itself but serves as a precursor to form biologically active molecules, including 5-oxo-ETE, HKs, and 5-OH-PGs. 5-oxo-ETE is an oxidized metabolite of 5*S*-HETE that binds to the 5-oxo-ETE receptor (OXE), exerting a powerful granulocyte chemoattractant effect [60,61,62].

**Figure 5:**
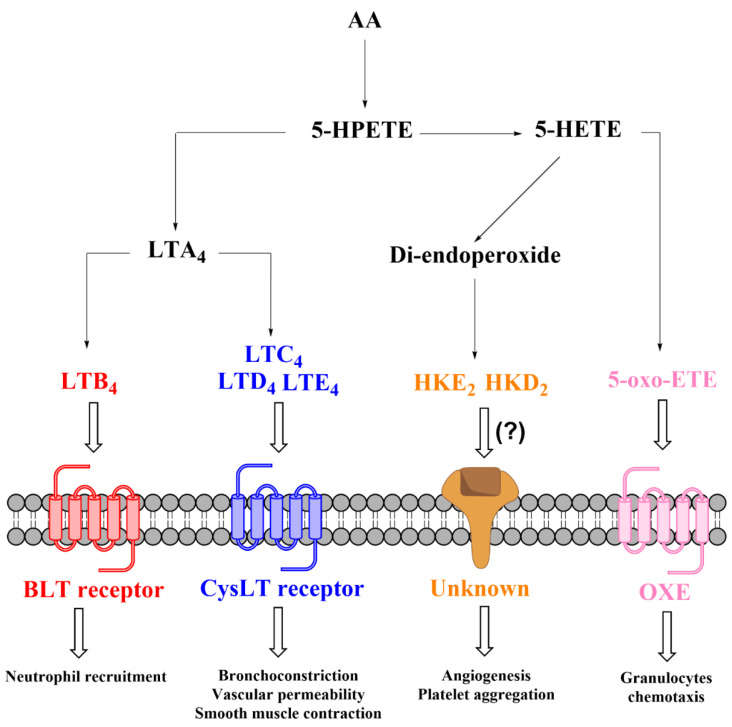
Biological effects of 5-LOX-derived eicosanoids.

The recently described biochemical and chemical synthesis of HKs [63,64] enables investigating the biological role(s) of these newly discovered eicosanoids. An established activity of these molecules is the stimulation of endothelial cell migration and tubulogenesis [13] and the modulation of platelet aggregation [63], implying a possible role in atherosclerosis and CVD. However, unlike LTs, it remains unexplored thus far whether HKE_2_ and HKD_2_ exert their effects via interaction with a specific receptor (Figure 5). Even less is known about the biological activity of 5-OH-PGs that appear not to activate traditional prostanoid receptors [44].

### 1.4. Inflammation as a Target of Dietary (Poly)phenols: Role of Their Bioavailability

Evidence from epidemiological and observational studies highlight diet as one of the cornerstones in preventing inflammatory diseases such as intestinal inflammation and cardiovascular diseases. Dietary patterns that include a high intake of fruits and vegetables, such as the Mediterranean diet as a source of high levels of phytochemicals, including dietary (poly)phenols, have been shown to significantly ameliorate inflammation [65,66,67]. In this regard, some clinical trials have provided evidence supporting the beneficial role of dietary (poly)phenols against chronic inflammatory diseases [68,69]. However, while numerous preclinical studies describe the anti-inflammatory effects of many (poly)phenolic compounds through the modulation of a plethora of cellular processes related to inflammation, the evidence of activity in humans remains unclear overall from a nutritional point of view, partly owing to the limited bioavailability of (poly)phenols [18,70].

The bioavailability of dietary (poly)phenols is essential for a better understanding of the anti-inflammatory effects of (poly)phenolic compounds and to design physiologically relevant studies to corroborate their potential effects. Plant-derived foods (e.g., *Citrus* fruits, walnuts, pomegranates, green tea, soy, grapes, and others) contain phenolic compounds in free form or conjugated with sugar moieties, which are not well absorbed in the small intestine, thus limiting their distribution in systemic tissues in their native form. Upon consumption, (poly)phenols reach the gastrointestinal tract in their original molecular form, mainly as glycosides and complex oligomeric structures, and are hydrolyzed and further metabolized by either intestinal enzymes or by the gut microbiota forming new metabolites [71,72]. For instance, ellagitannins (ETs) (such as punicalagin from pomegranate) and their hydrolysis product ellagic acid (EA) undergo gut microbiota metabolism to yield metabolites collectively termed urolithins (Uro), with the most relevant ones identified as Uro-C, Uro-A, IsoUro-A, and Uro-B. Similarly, isoflavones (IsoFlv) and their aglycones (e.g., daidzin and daidzein, respectively) also undergo microbial metabolism producing equol and(or) *O*-desmethyl-angolensin (ODMA) [71,73]. The flavonoid glycoside rutin (quercetin-3-rutinoside) acts as a precursor (via deglycosylation) of quercetin in the colon [74], whereas curcumin and flavanones (such as the glycoside hesperidin and its aglycone hesperetin) can be detected in the colon for hours in their original form [75,76]. Upon absorption, the (poly)phenolic compounds and the microbial metabolites undergo phase-II metabolism to form conjugated molecules (glucuronides, sulfates, and methyl esters), which are the main molecules detected in the bloodstream, intestinal tissues, bile, feces, urine, and different systemic tissues [77,78]. Animal and human metabolism and bioavailability studies have reported that, at the intestinal level, the parental phenolic compounds (which serve as substrates of the gut microbiota) and their microbial-derived metabolites can achieve concentrations from μM to mM. In contrast, the concentrations reached in the bloodstream by the phase-II metabolites can range from nM to low μM, show anti-inflammatory effects and persist in the circulation for a few days after intake [79]. Among the phase-II metabolites detected in vivo, glucuronides are recognized as the major conjugated molecules, including Uro-A glucuronide (Uro-A glur), Quercetin-3 glur, and curcumin-glur [80,81,82]. Interestingly, the increasing knowledge about the metabolism of these compounds indicates that, under inflammatory conditions, the circulating glucuronides might also play a role as precursors of their aglycones, including luteolin [83,84], quercetin [85,86,87,88,89], resveratrol [90], Uro-A [91], and curcumin [82]. Besides, a recent trial showed that the intake of a (poly)phenolic cocktail by breast cancer patients allowed detecting relevant concentrations of free curcumin, most likely due to a conjugation-saturation process, in malignant mammary tumors [77].

## 2. Methods

### Search Strategy and Study Selection

This review was conducted and reported following the Preferred Reporting Items for Systematic Review and Meta-Analyses (PRISMA) [92]. A comprehensive literature search was performed using PubMed and Scopus databases. The search strategy included the combination of the following search terms in abstracts and titles and was adapted for each database: (5-lipoxygenase or 5-LOX) and (phenol* or flavonoid* or polyphenol* or curcumin or resveratrol or EGCG or urolithin or procyanidin or proanthocyanidin or flavan* or flavone* or catechin or epicatechin or quercetin or curcumin or valerolactone or punicalagin or ellagic or tannin or lignan or isoflavone or equol or silymarin or thistle). Independent literature searches and article selection were completed from January to April 2021 by the authors. The authors also hand-searched the bibliography of identified articles.

Regarding eligibility criteria and study selection, all human, animal, and cellular model studies that investigated the role of 5-LOX in the anti-inflammatory effect of (poly)phenolic compounds were included in this review. Otherwise, enzymatic, in silico and in vitro studies that used physiologically unrealistic conditions, such as high concentrations (compared to those reported in vivo), irrelevant metabolic forms of (poly)phenols, inappropriate cellular models, and those conducted with plant extracts were excluded.

**Figure 6:**
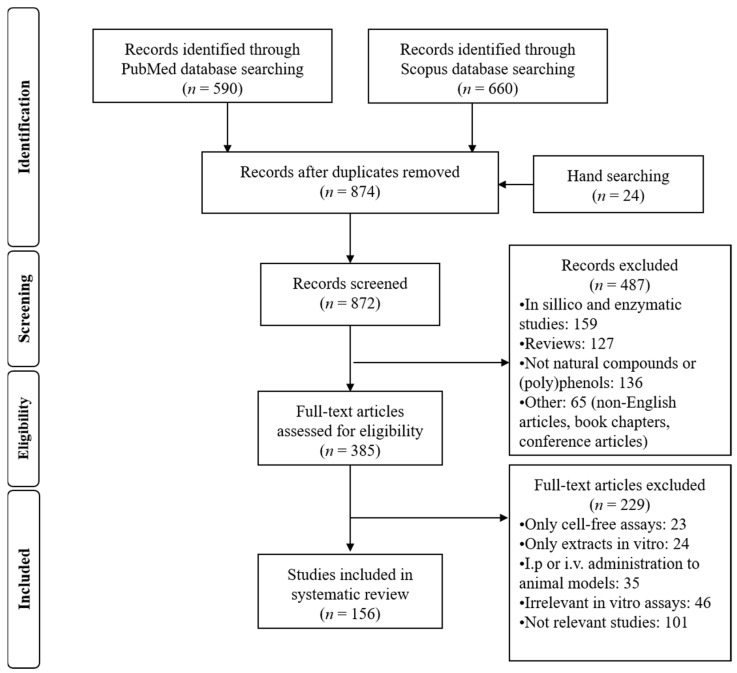
PRISMA flow diagram. Description of the search strategy and exclusion/inclusion criteria.

A total of 1250 articles were found after the literature search in the two electronic databases. Removing duplicates and full screening yielded 872 articles, of which 5 human, 24 animal, and 127 in vitro cell model studies met the inclusion criteria for this systematic review. A summary of the selection of articles included in this study is outlined in Figure 6.

The selected studies are reviewed in detail in the following sections and are summarized in independent tables (Tables 1 and 2 and Table S1) to show the current evidence of the role of 5-LOX as a target of the anti-inflammatory effects of (poly)phenols.

## 3. Results and Discussion

### 3.1. Human Studies

Targeting 5-LOX to address inflammation using a nutritional approach has been tested in only a few human studies. Only five studies determined the level of 5-LOX-derived metabolites in subjects who consumed plant-derived foodstuff (Table 1). These studies are characterized by a small number of subjects (*n* = 10–18) and short-term duration (up to four weeks). Four investigations using healthy volunteers described the modulation of 5-LOX expression and its metabolites (LTs and lipoxins) after the consumption of soy milk, procyanidin-enriched chocolate bars, enriched beverages, or olive oil (Table 1). One human study evaluated the effect of a diet supplemented with soy isoflavone tablets in patients with asthma. Lung function parameters remained unaltered, while isolated eosinophils showed an attenuated capacity to synthesize LTB_4_ ex vivo after stimulation [93]. The absence of a noticeable effect of (possible) modulation of LTs by polyphenolics in healthy subjects can be somewhat predicted and is in accord with 5-LOX-deficient animals, which are largely normal in the absence of an inflammatory stimulus [49]. Therefore, an insufficient number of human studies, mostly conducted in healthy subjects, makes it difficult to draw conclusions or even speculate regarding the beneficial effects of dietary phenolics via modulation of the 5-LOX pathway in humans.

### 3.2. Animal Studies

Table 2 summarizes animal studies that considered 5-LOX (together with other markers) a potential target of plant extracts and phenolic compounds administered through the diet.

Overall, a common anti-inflammatory effect observed in animals fed diets enriched with distinct plant extracts was the reduction of the carrageenan-induced paw edema volume as a prototypical assay to determine anti-inflammatory activity in vivo. Further exploration of the underlying molecular mechanisms showed inhibition of 5-LOX and COX-2 activities, lower LTB_4_ and PGE_2_ levels, and modulation of related inflammatory markers. Studies using animal models of arthritis described positive effects by ethanolic extracts of *Jasminum laceolarium, Vitex negundo*, *Dendropanax dentiger*, and *Pterospermun heterophyllum* [98,99,100,101] linked to the modulation of 5-LOX and COX-2 expression in PBMCs. However, it is unclear what compound(s) were responsible for the benefits described since crude extracts were used and the composition of the extracts was unexplored.

The consumption of diets supplemented with phenolic compounds (single or mixtures) showed beneficial anti-inflammatory effects via modulation of the 5-LOX pathway. Among the phenolic compounds, curcumin inhibited 5-LOX activity in PMNLs and reduced LTC_4_ biosynthesis in paw edema (alone or combined with capsaicin) and in animal models of anaphylaxis [102,103]. Caffeic acid ameliorated brain and liver damage via down-regulation of 5-LOX mRNA expression and protein level [104,105]. Flavocoxid, a dual inhibitor of 5-LOX and COX-2, targeted 5-LOX at the intestinal level, reducing LTB_4_ levels and MPO activity (exerted by neutrophils) in colitis animal models [106]. As shown in Table 2, other phenolics such as catechins, quercetin, salidroside, nordihydroguaiaretic acid (NDGA), sesamol, and sesamin alleviated induced inflammation through similar molecular mechanisms, including 5-LOX-mediated inflammation.

### 3.3. In Vitro Studies

In vitro cell models are an essential tool to investigate the underlying molecular mechanisms by which phenolic compounds exert their effects. Numerous in vitro studies indicate that a wide range of phenolic compounds might exert anti-inflammatory effects by targeting the 5-LOX pathway (Table S1). Thus, caffeic acid, hydroxytyrosol, resveratrol, curcumin, NDGA and quercetin are compounds with the capacity to reduce the formation of 5-HETE, LTB_4_ (and its ω-oxidized metabolites), and Cys-LTs [107,122,123,124,125,126,127,128,129,130,131,132,133,134,135,136,137,138,139,140]. Although desirable, these effects should be considered with caution since inhibition of the 5-LOX pathway could result in higher (pro-inflammatory) COX-2 metabolite levels by shunting the substrate, AA. This phenomenon has been described in stimulated leukocytes and mast cells treated with caffeic acid and NDGA, respectively [141,142].

Blood- and peritoneal-isolated leukocytes are a widely employed cellular model, allowing to determine whether the phenolic compounds exert dual inhibition on 5-LOX and COX-2. In general, some phenolic compounds such as curcumin, resveratrol, and caffeic acid show the capacity to attenuate the biosynthesis of 5-LOX (5-HETE, LTB_4_, Cys-LTs) and COX-2 (PGE_2_) metabolites in stimulated leukocytes (Table S1). As shown in Table S1, different molecular mechanisms account for how phenolic compounds modulate the biosynthesis of eicosanoids. One of these mechanisms involves the interaction with the upstream regulator cPLA_2_. This interaction can include lower levels of cPLA_2_ (i.e., through down-regulation of mRNA expression), reduced activation (phosphorylation) or translocation, and inhibition of its enzymatic activity. Further mechanisms of action are related to the inhibition of 5-LOX translocation to the nuclear membrane where it becomes active, down-regulation of 5-LOX (and COX-2) mRNA and protein expression, as well as inhibition of 5-LOX (and COX-2) enzymatic activity (Table S1). Despite valuable results gained from these studies, other aspects of how phenolic compounds modulate the 5-LOX pathway are still unclear. For example, information on the interaction of phenolic compounds with (other) essential components of the 5-LOX pathway is scarce. None of the studies included in Table S1 tested whether the phenolic compounds target LT receptors (i.e., BLT1 and BLT2), FLAP, and/or LTC_4_ hydrolase. Regarding LTA_4_ hydrolase, only one study showed that isoflavones failed to inhibit (or promote) its enzymatic activity [143]. Thus, the characterization of the interaction of phenolic compounds with these elements will be essential in future studies.

Studies using single-cell lines provide essential information about how the phenolic compounds modulate 5-LOX (and COX-2) activity in different leukocytes. Curcumin reduced the formation of LTB_4_ and PGE_2_ in neutrophils [144] and macrophages [145]. Curcumin also targeted LTC_4_ biosynthesis in mast cells by blocking 5-LOX translocation and cPLA_2_ activation [102]. Quercetin reduced biosynthesis of LTB_4_ in RBL-1 basophils, neutrophils, and murine PB-3c mast cells [146,147,148], while genistein acted on eosinophils decreasing LTC_4_ synthesis via inhibition of 5-LOX translocation [95]. Likewise, the effect of silibinin on macrophages (Kupffer cells) was associated with lower LTB_4_ levels while sparing the COX-2 pathway [149,150].

A common facet to the majority of in vitro studies (Table S1) is the treatment of leukocytes with glycosides and/or aglycones using conceivable concentrations at the intestinal level. Such conditions indicate that the effects observed on eicosanoid biosynthesis might be relevant in the context of intestinal inflammation [151]. These studies focused on describing the effects on leukocytes, thus overlooking the crucial role of other cells (i.e., intestinal cells). To date, a limited number of studies have investigated the interaction between dietary phenolics and the 5-LOX and COX-2 pathways in intestinal cells. In this regard, NDGA and geraniin (at 10 μM) decreased the synthesis of 5-HETE in stimulated AGS cells [152], whereas methoxy flavonoids isolated from *Chiliadenus montanus* failed to modulate the expression of 5-LOX in Caco-2 cells [153]. The effects of curcumin and tetrahydrocurcumin (THC) on HT-29 colon cancer cells were related to lower AA levels associated with cPLA2 inhibition; yet, how this affected the 5-LOX (or COX-2) pathway was not determined [145].

Leukocytes are abundant in the bloodstream, making them an excellent model to test in vitro the anti-inflammatory effects of phase-II metabolites. However, according to our analysis, only two studies have approached the effect of conjugated metabolites on 5-LOX and COX-2 using leukocytes (Table S1). Among the conjugated metabolites tested, 3′-*O*-methyl-quercetin reduced the biosynthesis of LTB_4_ (at 2 μM) and PGE_2_ (IC_50_ = 2 μM) [154], whereas the phase II conjugated urolithins (ellagic acid-derived metabolites) were inactive against 5-LOX, COX-2, and the 5-LOX/COX-2 crossover pathways [155]. Under the same conditions, their free forms (quercetin, Uro-A, IsoUro-A, and Uro-C) effectively decreased 5-HETE, LTB_4_, PGE_2_, HKE_2_, and HKD_2_ levels in a dose-dependent manner. Although the free forms are hardly found in the bloodstream, their presence in inflammatory environments is conceivable via deconjugation, as described for luteolin, quercetin, resveratrol, Uro-A, and curcumin [77,82,83,84,85,86,87,88,89,90,91].

To date, no studies describe the in vitro effect of phenolic compounds or derived metabolites on lipoxin biosynthesis. Only two studies have investigated the effect of phenolics on the 5-LOX/COX-2 crossover pathway and found that curcumin and urolithins (Uro-A, IsoUro-A, and Uro-C) showed the capacity to inhibit HKE_2_ and HKD_2_ formation in a mixture of stimulated leukocytes [47,155]. This limited evidence underscores the need for future studies on the biosynthesis of complex 5-LOX dependent eicosanoids using isolated cell preparation or co-incubations (i.e., platelets with neutrophils) in the presence of phenolic compounds.

## 4. Conclusions

This systematic review focuses on the effect of phenolic compounds on the 5-LOX pathway. The current evidence linking modulation of the 5-LOX pathway and the anti-inflammatory effects of phenolic compounds is still weak. One of the main reasons comes from the low number of human studies and clinical trials, which are essential to test the preventive and/or therapeutic effects of phenolic compounds. Thus, well-designed and robust clinical trials in patients suffering from 5-LOX-related inflammatory diseases (e.g., asthma) would be desirable. The number of animal studies is also small and more research is needed using equivalent conditions to those reported in humans, including adequate exposure times and doses of phenolic-rich foodstuff.

As expected, a higher number of in vitro studies describe the anti-inflammatory effects of phenolic compounds targeting the 5-LOX pathway. The in vitro studies focus on using mixtures of leukocytes or individual cells such as neutrophils, eosinophils, basophils, and/or mast cells. New studies should consider enlarging the range of 5-LOX-expressing cells such as dendritic cells and should consider the interaction with other cell lines such as endothelial (i.e., atherosclerosis) or intestinal cells and microbiota (i.e., intestinal inflammation). The specific role of immune-related receptors regarding the modulation of the 5-LOX pathway by phenolic compounds is an additional mechanism (not considered hitherto) that deserves attention, such as the direct effect of phenolic compounds on LT receptors or via modulation of TLR-4, which regulates CysLT1 expression in dendritic cells [156]. These studies should avoid the use of unreasonable concentrations and metabolic forms of phenolic compounds (considering metabolism and bioavailability) since this inadequate design limits the physiological significance (from an in vivo point of view) of these investigations. Hence, the design of future in vitro studies should follow the roadmap set elsewhere [157] to provide physiologically relevant results.

Another key point to contemplate is 5-LOX/COX-2 dual inhibition. Targeting the 5-LOX pathway might lead to undesirable side effects due to AA shunting towards pro-inflammatory COX-2-derived metabolites. Therefore, identifying and studying the biological activity of phenolic compounds that act as dual inhibitors is critical to avoid single inhibition and resulting side effects. Otherwise, a possible drawback of this approach is the reduced biosynthesis of anti-inflammatory eicosanoids due to blocking cyclooxygenases and lipoxygenases (including 5-LOX). Whether the phenolic compounds can exert anti-inflammatory effects by increasing the synthesis of lipoxins is a question not addressed by the available preclinical studies. The expanded analysis of pro- and anti-inflammatory eicosanoids in future studies will improve the understanding of how phenolic compounds modulate the inflammatory response through the 5-LOX pathway.

## Figures and Tables

**Table 1:** Human studies describing the effects of the consumption of (poly)phenols on 5-LOX and its products.

Population of Study	Design of the Study	Foodstuff; Intake and Duration	Main Outcomes	Ref.
Healthy volunteers (*n* = 10; 20–55 years)	Randomized, crossover, double-blind, placebo-controlled.	Low- (0.09 mg/g) and high-procyanidin (4.0 mg/g) chocolate bars; 37 g (single dose); Duration: blood collection at 2 and 6 h; wash-out period of 1 week between treatments.	↑Epicatechin plasma level (especially the high procyanidins consumer group). ↓CysLTs/PGI_2_ ratio (relative to the effects observed in the low procyanidins consumer group).	[94]
Patients suffering mild/moderate persistent asthma (*n* = 13; 18–65 years)	Quasi-experimental intervention study. No control group.	^1^ Soy isoflavones tablets (NOVASOY, ArcherDanielsMidland, Decatur, IL, USA); two 50 mg tablets (once per day); duration: 4 weeks.	↓LTB_4_ and FE_NO_ in A23187-stimulated eosinophils (ex vivo); no significant changes in pre-bronchodilator FEV_1_ and Juniper Asthma Control Score.	[95]
Healthy volunteers (*n* = 18; 22–44 years)	Acute consumption, no control group.	Raw virgin olive oil; 50 mL (single dose consumed with 200 g bread); Duration: blood collection at 1 and 6 h; 1 week wash-out period before olive oil consumption.	↑Hydroxytyrosol in plasma. ↓ALOX5AP gene expression at 1 h (basal values at 6 h). The downregulation was inversely correlated with glucose and insulin levels.	[96]
Healthy volunteers of Asian ethnicities (*n* = 18)	Randomized, crossover, double-blind, placebo-controlled.	^2^ Soy milk; 2 daily treatments (20 g); Duration: 3 h after acute consumption followed by 1-week wash-out period and 4-weeks daily exposure.	↓LTB_4_ and LXA_4_ level in plasma after 3 h; ↓LTB_4_ and F_2_-isoprostanes in plasma and urine after 4-weeks daily exposure; ↑LXA_4_ in plasma after 4-weeks daily exposure; ↓MPO activity, serum lipid hydroperoxides and hsCRP in plasma after 4-weeks daily exposure.	[93]
Senior and young taekwondo athletes (*n* = 10; 18–57 years old)	Quasi-experimental intervention study, no placebo.	Isotonic beverage enriched with almond (0.3%), sucrose (0.8%), oils (0.2% ^3^ DHA-S and 0.6% olive oil), and α-tocopherol acetate (vitamin E); duration: 5 weeks (consumed 5 days a week); blood samples taken at the beginning and at the final of the (1 h before and after each stress test).	Beverage supplementation exerted ↓sL-Selectin, sICAM3 and ↑IL-6 in young athletes (after exercise) as well as ↑TNF-α level in plasma in the young group (in absence of exercise) and in the senior group (after exercise). The beverage consumption exerted no significant effects on lipoxin, PGE_2_, PGE_1_, and NF-κB. Modulation of TNF-α, 15-LOX2, COX-2, IL-1β and IL-8 mRNA expression in PBMC; No Effect on TLR_4_, NF-κB, 5-LOX, IL-10, IL-15, HSP72 expression (mRNA) in PBMC.	[97]

Abbreviations: CysLTs, cysteinyl leukotrienes; DHA, docosahexaenoic acid; FE_NO_, fraction of exhaled nitric oxide; FEV_1_, forced expiratory volume; LTB_4_, leukotriene B_4_; LXA_4_, lipoxin A_4_; HSP72, heat shock protein 72; hsCRP, high sensitivity C-reactive protein; MPO, myeloperoxidase; sICAM3, soluble intracellular adhesion molecule 3; NF-κB, nuclear factor kappa-light-chain-enhancer of activated B cells; PBMC, peripheral blood mononuclear cells; PGI_2_, prostacyclin; TLR-4, toll-like receptor-4; TNF-α, tumor necrosis factor- α, Composition: ^1^ Soy isoflavones tables: 8 mg glycitein, 28 mg daidzein, 29 mg genistein. ^2^ Soy milk powder with the BASF Vegapure^®^: stigmasterol, campesterol, and palmitates of β-sitosterol (maximun 23% *w*/*w*, 29% *w*/*w*, 55% *w*/*w*, respectively); ^3^ DHA-S; nutritional oil obtained from *Schizochitrium* sp. (marine alga).

**Table 2:** In vivo studies carried out with (poly)phenols-rich extracts or individual compounds in relation to inflammation and 5-LOX modulation.

Animal Model	Extract/Compound Assayed	Dose/Duration	Main Outcomes	Ref.
♂ Wistar rats; carrageenin-soaked sponges implanted subcutaneously.	Quercetin and NDGA.	100 mg/kg; administered (16 and) 1 h prior sponge implantation.	No effect on leukocyte infiltration, oedema formation or PGE_2_ and LTB_4_ formation in A23187-stimulated leukocytes ex vivo.	[107]
♂ Wistar rats; ethanol-induced gastric mucosal damage.	NDGA.	100 mg/kg (prepared in 0.25% carboxymethylcellulose); administration of a single dose for 30 min.	↓Gastric lesions and LTC_4_ biosynthesis; no changes on PGs and TxB_2_ production.	[108]
♂ Sprague Dawley rats; cadmium-poisoned rats.	Crude catechin powder. ^1^	0.25 and 0.5 g powder/100 g diet; 4 and 20 weeks.	Assays in platelets (ex vivo): ↓PLA_2_ and COX-1 activity, ↓TxB_2_; assays in aortic slices: ↓6-keto-PGF_1α_ and ↓LTB_4_ in A23187-stimulated leukocytes; ↓5-LOX activity (enzyme fraction level).	[109,110]
♂ Wistar rats; carrageenan-induced paw inflammation.	Curcumin, capsaicin, and a mix of curcumin/capsaicin.	Curcumin: 0.2%, capsaicin: 0.015%, curcumin/capsaicin: 0.2/0.015%; 10 weeks.	↓Volume of paw edema; ↓5-LOX activity in the enzyme obtained from PMNL isolated from blood of the rats; ↓histamine release.	[103]
♂ KM strain mice; aluminum-induced brain damage.	Caffeic acid.	10 or 30 mg/kg; days.	↓5-LOX mRNA expression in the cortical brain (at 10 and 30 mg/kg) and protein expression in hippocampi (only at 30 mg/kg); improvement of memory and learning functions together with ↓MDA, ↓ChAT, and ↓amyloid β and amyloid precursor protein.	[105]
♂ Wistar rats; carrageenan-induced paw edema.	*Bacopa monniera* extracts.	20–200 mg/kg; 3 and 5 h.	↓Volume of paw edema; ↓LPS-induced TNF-α release in whole blood (ex vivo); ↓5-LOX and 15-LOX (IC_50_ = 100 μg/mL) as well as COX-1 (IC_50_ = 15.66 μg/mL) and COX-2 (IC_50_ = 1.22 μg) in A23187-induced rat mononuclear cells (ex vivo).	[111]
♀ New Zealand white rabbits; hypercholesterolemic diet.	Quercetin.	25 mg/kg; 90 days.	↓5-LOX, 12-LOX, COX, activity in rabbit mononuclear cells; ↓CRP in plasma, ↓MPO activity in the aorta, and improvement of lipid profile and histopathological aortic features.	[112]
♂ Wistar albino rats; carrageenan-induced paw edema.	*Atropa acuminata* ethanolic extract.	62.5–500 mg/kg b.w.; up to 4 h.	↓LTB_4_ and PGE_2_ in carrageenan-treated paws; ↓leukocyte and neutrophil recruitment (no effect on mononuclear cells); ↓vascular permeability; ↓paw edema and exudate volume; modulation of the antioxidant status.	[113]
ICR mice; IgE/Ag-mediated passive systemic anaphylaxis.	Curcumin.	20 and 50 mg/kg; 1 h.	↓LTC_4_, PGD_2_ and histamine.	[102]
♂ Wistar rats; CFA-induced rheumatoid arthritis.	*Xanthium strumarium* extract.	75 and 300 mg/kg; administered twice a day after the adjuvant arthritis induction for 28 days.	↓5-LOX and COX-2 expression in PBMCs; ↓paw swelling and arthritic score; ↓TNF-α and IL-1β together with ↑IL-10 in serum; improvement of histopathological features.	[114]
♂ Wistar albino rats; CFA-induced arthritis.	*Vitex negundo* seed extract.	85 and 340 mg/kg/day; 28 days.	↓Paw swelling (from day 14^th^) and clinical arthritis score; attenuation of CFA-induced weight loss and index of spleen; ↓synovial lining hyperplasia and massive infiltration of mononuclear cells; ↓TNF-α, IL-1β (at both concentrations) and IL-6 (at 340 mg/kg); ↑IL-10; ↓COX-2 and 5-LOX expression in isolated PBMC.	[101]
♂ Sprague Dawley albino rats; Isoproterenol-induced myocardial infarction.	*Ocimum sanctum* methanolic extract.	50–250 mg/kg b.w.; 30 days.	↓TBARS and NF-κB expression in the heart; ↓FLAP and BLT1 (mRNA) expression in the heart; ↓PLA, PLC and PLD activity, whereas ↑SOD activity and phospholipids in the heart; ↓CK-MB, LDH, hsCRP, LTB_4_, TxB_2_ (in serum); ↓COX-2 and 5-LOX activity in monocytes; attenuation of the effects of isoproterenol on cardiomyocytes.	[115]
♂ BALB/c mice; ethanol-induced gastric ulcer.	Salidroside.	20 and 40 mg/kg; 6 days.	↓5-LOX and COX-2 protein expression; ↓LTB_4_ level; modulation of the MAPK and NF-κB pathways; ↓IL-6, IL-1β and TNF-α; improvement of gastric histopathological features.	[116]
♂ Wistar rats; carrageenan-induced rat paw edema.	*Jasminum laceolarium*.	100–400 mg/kg; 7 days.	↓5-LOX (only at 400 mg/kg) and COX-2 expression in serum; ↓paw edema.	[98]
♀ Sprague Dawley albino rats; HCD-fed atherosclerotic rats.	Quercetin.	25 mg/kg b.w.; 60 days.	↓5-LOX and COX activity as well as IL-6 expression (mRNA) in mononuclear cells; ↓NOS activity and CRP in serum; ↓MDA in serum and aorta.	[117]
♂ Sprague Dawley rats; DNBS- and DSS-induced colitis.	Flavocoxid.	20 mg/kg/day (twice a day); 4–5 days.	↓LTB_4_, PGE_2_, 6-keto PGF_1α_, TxB_2_, and TNF-α serum level; ↓MPO activity and MDA level in colon tissue; ↓histological damage and apoptosis; ↓CD3 in colon tissue.	[106]
♂ Wistar rats; LPS-induced inflammation.	Sesamol, sesamin, and a mix of sesamol/sesamin.	10 mg/kg b.w.; 15 days.	↓LTB_4_, LTC_4_, MCP-1, IL-1β, CRP, and TNF-α serum level; ↓5-LOX, cPLA_2_, and BLT-1 protein expression; ↓LTC_4_ synthase protein expression (only sesamol and sesamin); ↓MDA (liver tissue and serum); ↓NO serum level (only sesamol); modulation of the antioxidant enzymes.	[118]
♂ Wistar rats and New Zealand rabbits; MSU crystal-induced inflammation.	Salidroside.	40–80 mg/kg for rats and 20–40 mg/kg for rabbits; 6 days.	↓LTB_4_, PGE_2_, and 20-HETE level in synovial fluid macrophages; ↓COX-2, 5-LOX, and CYP4A1 mRNA expression (only at 80 mg/kg) in synovial fluid macrophages; ↓number of leukocytes and neutrophils; binding to the catalytic side of 5-LOX, COX-2, and CYP4A1 (in silico); ↑macrophages polarization; improvement of ankle swelling and histopathological features.	[119]
♂ Wistar rats and Swiss albino mice; carrageenan-inflammation model and acetic acid-induced writhes.	*Salix tetrasperma* methanolic extract.	200–600 mg/kg; single dose.	↓COX-2, 5-LOX, PGE_2_, TNF-α, iNOS level, and NF-κB activation in sciatic nerve and brain stem; ↓oxidative stress; ↓p53 positive cells in brain stem tissue; ↓paw edema in rats and leukocyte migration in mice; ↓acetic acid-induced writhes; ↑response latency to heat hyperalgesic stimulus; improvement of histopathological features; antipyretic effect.	[120]
♂ Sprague Dawley rats; aluminum gluconate-induced liver injury.	Caffeic acid.	30 mg/kg.	↓5-LOX protein expression in the liver (no effect on COX-2); ↓TNF-α, IL-1β, IL-6, MDA and ↑SOD in the liver; improvement of histopathological features; modulation of the alteration of hepatic enzymes;	[104]
♂ Sprague Dawley rats; adjuvant-induced arthritis.	*Pterospermun heterophyllum* ethanolic extract.	160–640 mg/kg/day; 22 days.	↓5-LOX, COX-2, and MMP-2 expression in rat-isolated PBMCs; ↓TNF-α, IL-1β, IL-6, IL-17, RF, and CRP serum level; ↑IL-4 and IL-10 serum level; improvement of histopathological features of the knee joint and arthritis markers.	[100]
♂ Sprague Dawley rats; adjuvant-induced arthritis.	*Dendropanax dentiger* ethanolic extract.	127.5–510 mg/kg/day; 22 days.	↓5-LOX, COX-2, and MMP-2 expression in rat-isolated PBMCs; ↓TNF-α, IL-1β, IL-6, IL-17, RF, and CRP serum level; ↑IL-4 and IL-10 serum level; improvement of histopathological features of the knee joint and arthritis markers.	[99]
♂ Sprague Dawley rats; pharmaco-kinetic study. ♀ Wistar rats; MIA-induced knee OA.	A curcumin formulation (NGUC) or turmeric extract.	NGUC or 95% turmeric extract as 100% (w/v) aqueous solution to deliver 200 mg/kg b.w. equivalent of curcuminoids.	Enhanced total curcuminoids bioavailability in NGUC-treated animals; reduced swelling; improvement of joint architecture; ↓IL-6, IL-1β, TNF-α, CRP, COMP, NF-κB, COX-2, MMP-3, 5-LOX, COX-2 in synovial fluid; ↓MDA, SOD, CAT, and GPx level.	[121]

Abbreviations: AA, arachidonic acid; BLT-1, leukotriene B_4_ receptor-1; CAT, catalase; ChAT, choline acetyltransferase; CFA, complete Freund’s adjuvant; CK-MB, creatinine kinase-MB; COMP, cartilage oligomeric matrix protein; cPLA2, cytoplasmic phospholipase A_2_, CRP, C-reactive protein; DNBS, dinitrobenzenesulfonic acid; DSS, dextran sulphate sodium; EpiCat, epicatechin; EpiGal, epigallocatechin; FLAP, 5-lipoxygenase-activating protein; GPx, glutathione peroxidase; HCD, hypercholesterolemic diet; HETE, hydroxyeicosatetraenoic; hsCRP, high sensitive C-reactive protein; iNOS, inducible nitric oxide synthase; LDH, lactate dehydrogenase; LPS, lipopolysaccharide; MAPK, mitogen activated protein kinase; MCP-1, monocyte chemoattractant protein-1; MDA, malondialdehyde; MIA, monosodium iodoacetate; MMP, matrix metalloproteinase; MPO, myeloperoxidase; MSU, monosodium urate; NDGA, nordihydroguiaretic acid; NF-κB, nuclear factor kappa-light-chain-enhancer of activated B cells; NGUC, next generation ultrasol curcumin; NO, nitric oxide; OA, osteoarthritis; PBMCs, peripheral blood mononuclear cells; PLA, phospholipase A; PLA_2_, phospholipase-A_2_; PLC, phospholipase C; PLD, phospholipase D; RF, rheumatoid factor; SOD, superoxide dismutase; STZ, streptozocin; TBARS, thiobarbituric acid reactive substance; TNF-α, tumor necrosis factor-α; TxB_2_, thromboxane B_2._ Composition: ^1^ Crude catechin powder from green tea: 4.56% EpiGal, 4.52% EpiCat, 38.56% EpiGal gallate, 20.76% EpiCat gallate.

## Supplementary Materials

The following are available online at https://www.mdpi.com/article/10.3390/ijms22157937/s1, Table S1: Modulation of 5-LOX activity and comparison with COX-2 activity by (poly)phenols in cellular models.
