# Supplementary material for: Targeting Mammalian 5-Lipoxygenase by Dietary Phenolics as an Anti-Inflammatory Mechanism: A Systematic Review
Source: Int J Mol Sci. 2021 Jul 25;22(15):7937. doi: 10.3390/ijms22157937 (PMC8348464; doi:10.3390/ijms22157937)
Supplement: Supplementary file 1 [file ijms-22-07937-s001.zip › ijms-1293418-supplementary.pdf]

International Journal of
Molecular Sciences

MDPI

Table S1. Modulation of 5-LOX activity and comparison with COX-2 activity by (poly)phenols in cellular models. 1

Cellular model	Metabolite and assay conditions	Biological activity on 5-LOX and(or) COX-2 and molecular associated events	Effect on 5-LOX enzyme	Effect on COX-2 enzyme	References
Guinea pig isolated leukocytes	NDGA (0.5 – 30 μM)	↓LTB_4, 5-HETE and di-hydroxy acids formation (IC_{50} ~ 2 μM) in A23187-stimulated cells in a dose-dependent manner	Not evaluated	Not evaluated	[1]
Isolated peritoneal mouse macrophages (prelabelled with [^{14}C]-arachidonic acid) and rat neutrophils. Cell-fee assays with soybean 15-LOX were also carried out.	Quercetin and NDGA (0.01-100 μM).	Effects in A23187-stimulated rat neutrophils: ↓LTB_4 and 5-HETE formation in cells treated with quercetin (IC_{50} = 6.2 μM) and NDGA (IC_{50} = 1 μM); ↓PGE_2 and TxB_2 synthesis in cells treated with quercetin (IC_{50} = 6.2 and 18.0 μM, respectively) and NDGA (IC_{50} = 42.0 and 17.6 μM, respectively).<br>Effects in zymosan-stimulated mouse macrophages: ↓PGE_2 and 6-Keto-$PGF_{1\alpha}$ formation in cells treated with quercetin (IC_{50} = 8.2 and 8.5 μM, respectively) and NDGA (IC_{50} = 2.4 and 4.2 μM, respectively).<br>Quercetin and NDGA inhibited soybean 15-LOX activity (IC_{50} = 3.2 and 3.6 μM, respectively) in a cell-free assay.	Not evaluated.	Not evaluated.	[2]
Mouse mast tumor cells and rat-isolated platelets. Enzymatic assays with 5-LOX and	Caffeic acid and its phenethyl ester (CAPE) isolated from	↓LTC_4 and LTD_4 synthesis (only caffeic acid at 10 and 100 μM); ↓AA-	↓5-LOX activity by caffeic acid (IC_{50} = 3.7) and	↑COX-2 activity by caffeic acid	[3]

COX-2 obtained from cloned mastocytoma P-815,2-E-6 cells were also carried out.	*Artemisia rubripes* Nakai (0.1-100 μM).	induced platelet aggregation; ↓12-HETE formation (IC_{50} = 30 μM); no effect on HHT and TxB_2 formation at <10 μM, but at higher doses (100 μM) exerted an inhibitory effect (40% HHT and 80% 12-HETE); no effect on ADP-induced platelet aggregation.	CAPE (IC_{50} = 0.48 μM)	(64%) and CAPE (188%)	
Human and porcine leukocytes and platelets. COX activity was tested in microsomes from ram vesicular glands.	6,7,4'-trihydroxyisoflavan (1 μM – 1 mM).	↓5-HETE, 12-HETE, and LTB_4 formation, while sparing the production of HHT (15 μM) in activated (human and porcine) leukocytes; ↓12-HETE formation without inhibiting the synthesis of TxB_2 and HHT in platelets (at 100 μM); IC_{50} values for 12-LOX (human platelets) and 5-LOX (leukocytes) were 22.0 and 1.6 μM, respectively.	Not evaluated.	↓HHT, $PGF_{2\alpha}$, PGD_2, and PGE_2 synthesis in ram seminal vesicle microsomes (IC_{50} = 200.0 μM). Total inhibition at 1 mM.	[4]
Human isolated platelets and leukocytes	NDGA	↓5-HETE (IC_{50} = 0.2 – 0.3 μM), LTB_4 (IC_{50} = 0.5 μM), LTC_4 (IC_{50} = 0.2 – 0.5 μM), Δ^6-trans-LTB_4 (IC_{50} = 0.2 μM), ω-OH-LTB_4 (IC_{50} = 2 μM), 12-HETE (IC_{50} = 5 – 30 μM), 5*S*,12*S*-diHETE (IC_{50} = 0.2 μM), 15-HETE (IC_{50} = 30 μM) and HHT (IC_{50} = 5 – 100 μM)	Not evaluated	Not evaluated	[5]
Human isolated platelets and leukocytes	NDGA (0.1 – 100 μM)	↓5-HETE, LTB_4, LTC_4 and 12-HETE	Not evaluated	Not evaluated	[6]
Human-isolated PMNL from healthy donors	Caffeic acid (1 μM) and esculetin (10 μM)	↓5-HPETE and 5-HETE release in Streptolysin 0-stimulated PMNLs.	Not evaluated.	Not evaluated.	[7]

Leishmania donovani-infected macrophages	NDGA (3 μM)	↓5-HETE and 12- or 15-HETE in infected cells	Not evaluated	Not evaluated	[8]
Human isolated leukocytes	NDGA	↓5-HETE (IC_{50} = 0.6 μM) and weak effect on PGE_2 formation (IC_{50} = 119 μM) in A23187-stimulated cells	Not evaluated	Not evaluated	[9]
Human colonic mucosa	NDGA (10 μg/mL)	↓sulfidopeptide-LTs in A23187-stimulated human colonic mucosa; no significant effect on PGE_2 formation	Not evaluated	Not evaluated	[10]
Human isolated neutrophils	NDGA (10 μM)	↓LTB_4 and 5-HETE formation in A23187-stimulated neutrophils; no effect on HHT synthesis	Not evaluated	Not evaluated	[11]
Human isolated leukocytes.	Gingerdione, [6]-gingerol, and curcumin.	↓5-HETE release by [6]-gingerol (14% at 20 μM) as well as by shogaol, capsaicin, gingerdione and curcumin (IC_{50} = 23, 100, 15.0 and 8.0 μM, respectively); ↓PGE_2 release (IC_{50} = 67, 73, 18.0, 68.0 and 52.0 μM for shogaol, capsaicin, gingerdione, [6]-gingerol, and curcumin, respectively) in A23187-activated leukocytes.	Not evaluated.	Not evaluated.	[12]
Human isolated PMNL	3,5-, 4,5-, and 3,4-di-*O*-caffeoylquinic acid, caffeoylmalic acid, caffeoyltartaric acid, rosmarinic acid, caffeic acid, and chlorogenic acid (1 μM – 1 mM).	↓LTB_4 and 5-HETE formation in A23187-stimulated PMNLs (IC_{50} = 55.6 – 92.5 μM and 214 – 918 μM for all compounds, respectively); ↓15-HETE formation only in the presence of rosmarinic acid (IC_{50} = 455 μM); ↑PGE_2 in the presence of 3,4-di-*O*-caffeoylquinic acid, caffeic acid, and caffeoylmalic acid; ↓HHT in the presence of the compounds tested (IC_{50} = 80.0 – 90.2 μM); chlorogenic acid was inactive.	Not evaluated.	Not evaluated.	[13]

Elicited rat peritoneal leukocytes.	Hypolaetin-8-glucoside, hypolaetin, and 14 other flavonoids (1 μM – 1 mM).	↓LTB_4 and TxB_2 production in A23187-stimulated leukocytes by various flavonoids. Glycosides were less active than their aglycones (i.e., hypolaetin-8-glucoside/hypolaetin; gossypin/gossypetin; rutin/quercetin; naringin/naringenin), with the exception of naringin/naringenin.<br>The flavonoids that stimulated DNA degradation also showed 5-LOX inhibitory activity, whereas those that lacked capacity to degrade DNA were related to COX inhibition.	Not evaluated.	Not evaluated.	[14,15]
Rabbit peritoneal PMNLs	Isoliquiritigenin (10 and 30 μM).	↓[^{14}C]5-HETE production in a concentration-dependent manner in A23187-stimulated PMNLs; no inhibitory effect on PLA_2 activity obtained from rabbit platelet sonicates; ↓[^{14}C]12-HETE formation in rabbit platelet sonicates.	Not evaluated.	Not evaluated.	[16]
Porcine leukocytes	Isoflavonoids such as isoflavans and isoflavones (0.5 – 200 μM)	↓5-LOX products (5-HETE, 5-HPETE, LTB_4 and stereoisomers of LTB_4); structure-activity investigation showed more effective inhibition in the presence of isoflavans	Not evaluated	Not evaluated	[17]
Rat peritoneal neutrophils and human platelets	Curcumin (1–50 μM).	↓LTB_4 synthesis in stimulated (calcium/A23187) rat peritoneal neutrophils (IC_{50} = 30 μM; NDGA = 0.5 μM). ↓COX-1 (IC_{50} = 2 μM) and 12-LOX activity (30 μM) in platelets.	Not evaluated.	Not evaluated.	[18]
Rat resident peritoneal macrophages.	Baicalein (1, 10 and 100 μM).	↓LTC_4 synthesis in A23187-stimulated macrophages (IC_{50} = 9.5 μM)	Not evaluated.	Not evaluated.	[19]

Murine resident peritoneal macrophages.	Genistein (1–100 μM).	↓PGE_2 production (IC_{50} = 20.0 μM) in response to zymosan, calcium ionophore A23187, and phorbol myristate acetate (PMA) stimulation; ↓LTC_4 production in response to zymosan and calcium ionophore A23187 (IC_{50} = 15.0 and 10.0 μM, respectively). These effects were not exerted via inhibition of PLA_2, COX or LOX enzymes.	Not evaluated.	Not evaluated.	[20]
Rat peritoneal leukocytes	Tanetin, fisetin, 3-hydroxy-flavone (1 – 100 μM)	↓LTB_4, TxB_2 and PGE_2 in A23187-stimulated cells in the presence of tanetin (IC_{50} = 11 and 6-11 μM for 5-LOX and COX-2, respectively), fisetin (IC_{50} = 11 and 50 μM for 5-LOX and COX-2, respectively) and 3-hydroxyflavone (IC_{50} = 80 and 6-8 μM for 5-LOX and COX-2, respectively)	Not evaluated	Not evaluated	[21]
Human isolated PMNL	RSV, piceid, 2,3,4′,5-tetrahydroxystilbene-2-*O*-D-glucoside, α-(3,4-dihydroxy-phenyl)-cinnamic acid, α-(3,4-dihydro-xy-phenyl)-3-hydroxycinnamic acid, α-(3,4-dihydro-xy-phenyl)-4-hydroxycinnamic acid, α-(3,4-dihydro-xy-phenyl)-3,4-dihydroxycinnamic acid, 3,3′,4-trihydroxystilbene	↓5-HETE, 5,12-diHETE, 15-HETE, HHT and PGE_2 in A23187-stimulated cells in the presence of RSV (IC_{50} = 8.9, 6.7, 275, 4.4, 9.6 μM, respectively) and 3,3′,4-trihydroxystilbene (IC_{50} = 5.9, 0.63, 6.77, 19, 49 μM, respectively). Piceid only affected 5-HETE (IC_{50} = 267 μM) and 5,12-diHETE (IC_{50} = 201 μM) biosynthesis; no effects observed in the presence of the rest of compounds<br>↓LTC_4 in A23187-stimulated cells in the presence of RSV (IC_{50} = 1.37 μM) and 3,3′,4-trihydroxystilbene (IC_{50} = 0.88 μM); ↓β-glucuronidase release	Not evaluated	Not evaluated	[22]

		and ↑cAMP concentration in the presence of RSV and 3,3′,4-trihydroxystilbene			
RBL-1 and RBL-2H3 cells	Honokiol	↓LTB_4 and LTC_4 formation in A23187-stimulated RBL-1 and IgE-treated RBL-2H3 cells; no effect on PLA_2, LTC_4 synthase and LTA_4 hydrolase activity	↓LTB_4 and LTC_4 in cell-free assays	Not evaluated	[23]
Isolated rat Kupffer cells and human phagocytic liver cells.	Silibinin (10, 25, 100 and 200 μM).	↓LTB_4 formation (IC_{50} = 15 μM) and no effect on PGE_2 formation in A23187-stimulated Kupffer cells. ↓Dose-dependent of O_2^- and NO production (IC_{50} = 80 μM). No effect on TNF-α formation. ↓LTB_4 formation in freshly isolated human phagocytic liver cells (10 and 25 μM).	Not evaluated.	Not evaluated.	[24]
Human platelets, white blood and endothelial cells.	Silibinin (1–25 μM to determine LOX products and 1–100 μM to determine COX products).	↓LTB_4 formation (IC_{50} = 15 μM) and Cys-LT formation (IC_{50} = 14.5 μM) in A23187-stimulated human granulocytes, but no effect on PGE_2 formation. ↓PGE_2 formation in LPS-activated human monocytes (IC_{50} = 45 μM). ↓TxB_2 and 6-keto-$PGF_{1\alpha}$ formation (IC_{50} = 69 and 52 μM, respectively), in human platelets and A23187-stimulated endothelial cells, respectively. ↓Hypochlorite production (IC_{50} = 7 μM), but not of O_2^- production in PMA-activated human granulocytes.	Not evaluated.	Not evaluated.	[25]

Rat platelets and rat PMNL	2-(3,4-Dihydroxy-phenyl) ethanol (DPE), 2-(4-Hydroxyphenyl) ethanol, 2-(3,4-dihydroxyphenyl) acetic acid, caffeic acid, protocatechuic acid, *p*-coumaric acid, *o*-coumaric acid, vanillic acid, and syringic acid (0.1 μM – 1 mM)	↓LTB_4 production in A23187-stimulated PMNLs and 12-HETE production in intact platelets (IC_{50} of DPE = 26.0 and 50.0 μM, respectively). DPE showed the highest inhibitory (compared with the rest of the compounds) activity against 5- and 12-LOX, especially in intact cells.	↓5-HETE synthesis (IC_{50} of DPE = 13.0) (5-LOX isolated from PMNLs)	↓12-HETE synthesis (IC_{50} of DPE = 4.2 μM), and no effect on TxB_2 synthesis (COX-1 and 12-LOX isolated from platelets)	[26]
PMNL from healthy donors.	Hydroxytyrosol (0.1 μM – 1 mM).	↓LTB_4 production (IC_{50} = 1.2 μM) and its ω-oxidized metabolites (20-hydroxy and 20-carboxy-LTB_4) in A23187-stimulated PMNLs	Not evaluated.	Not evaluated.	[27]
RBL-1 cells	YPE-01 (derived from yakuchinones), yakuchinone B, dimethyl-yakuchinone B; NDGA was used a control of 5-LOX inhibition	↓LTB4 and LTC4 biosynthesis in A23187-treated RBL-1 cells in the presence of YPE-01 (IC_{50} = 0.035 and 0.046 μM, respectively), yakuchinone B (IC_{50} = 0.49 and 0.61 μM, respectively), dimethyl-yakuchinone B (IC_{50} = 1.14 and 2.11 μM, respectively) and NDGA (IC_{50} = 0.27 and 0.5 μM, respectively)	↓5-LOX activity by YPE-01 (IC_{50} = 0.28 μM), yakuchinone B (IC_{50} = 0.37 μM), dimethyl-yakuchinone B (IC_{50} = 0.22 μM) and NDGA (IC_{50} = 0.49 μM) in cell-free assay	↓COX-1 and COX-2 activity by yakuchinone B and dimethyl-yakuchinone B in cell-free assays	[28]
Human PMNL from healthy donors.	*trans*-RSV (0.44 – 440 μM).	↓LTB_4, 6-trans- and 12-trans-epi-LTB_4 production in A23187-stimulated PMNLs (IC_{50} = 48.2±7.0 μM); formation of 5-LOX-derived metabolites was virtually abolished at 220 μM.	Not evaluated.	Not evaluated.	[29]
Bovine PMNL	Hyperoside, rutoside, and chlorogenic acid (25 – 100 μM).	No effect on LTB_4 or 12(*S*)-HETE biosynthesis at concentrations ≤100 μM in A23187-stimulated PMNLs.	Not evaluated.	Not evaluated.	[30]

Human isolated PMNLs and rat basophilic leukemia cells RBL-1	Myr-3-glur and NDGA	↓LTB_4 formation in A23187-stimulated PMNLs treated with Myr-3-glur for 2 h (IC_{50} = 2.2 μM) or with NDGA for 5 min (IC_{50} = 0.5 μM); no effect of Myr-3-glur in cells incubated for 5 min.<br>Myr-3-glur inhibited AA-induced platelet aggregation and ↓PGE_2, PGD_2, and PGI_2 biosynthesis ex vivo (to a similar extent as indomethacin)<br>↓COX-1 activity obtained from platelets (IC_{50} = 0.5 μM) and ram seminal vesicle (IC_{50} = 10 μM)	↓5-LOX activity in a crude enzyme preparation from RBL-1 (IC_{50} = 0.1 μM for Myr-3-glur and IC_{50} = 0.26 μM for NDGA)	↓COX-2 activity (IC_{50} = 0.26 μM) obtained from sheep placenta	[31]
Rat peritoneal leukocytes.	Gnaphalin, quercetin and galangin (20 – 160 μM).	Gnaphalin effects: ↓LTB_4 production in A23187-stimulated rat peritoneal leukocytes (IC_{50} = 81.8±12.9 μM; ↓TxB_2 production in rat peritoneal leukocytes stimulated with A23187 (IC_{50} = 39.9±3.9 μM), chemotactic peptide fMLP (IC_{50} = 12.0 μM) and AA (IC_{50} = 57.7±5.1 μM); no effect on the secretion of lysozyme, myeloperoxidase and<br>β-glucuronidase pro-inflammatory enzymes from neutrophil secretory granules; no scavenging effect against hydrogen peroxide or hypochlorous acid.<br>Quercetin effects: inhibition of LTB_4 (100%) and TxB_2 (55%) at 160 μM.<br>Galangin effects: inhibition of LTB_4 (11%) and TxB_2 (84%) at 40 μM.	Not evaluated.	Not evaluated.	[32]
Rat peritoneal leukocytes.	Oleuropein, tyrosol, hydroxytyrosol, and	↓LTB_4 production in A23187-stimulated rat peritoneal leukocytes treated	Not evaluated.	Not evaluated.	[33]

	caffeic acid (40, 100 and 200 μM).	with hydroxytyrosol, oleuropein, caffeic acid, and tyrosol (IC_{50} = 15, 80, 200, and 500 μM, respectively); ↓ROS production in PMA-stimulated rat leukocytes; no substantial inhibition on TxB_2 exerted by the compounds tested			
Human isolated PMNLs	3-methylcatechol, 4-methylcatechol, 4-nitrocatechol, guaiacol, pyrogallol, propylgallate, 1,2,4-trihydroxybenzene, 1,3,5-trihydroxybenzene (1.8 nM – 1800 μM)	↓LTB_4 (IC_{50} = 5 – 900 μM) and PGE_2 (IC_{50} = 45 – 900 μM) synthesis in A23187-stimulated cells (at concentrations higher than 18 μM); 1,3,5-trihydroxybenzene exerted no inhibition on LTB_4 formation (IC_{50} >> 1800 μM)	Not evaluated	Not evaluated	[34]
Human erythroleukemia K562 cells.	RSV (30 μM).	↓LTB_4 and PGE_2 synthesis in H_2O_2-treated K562 cells; ↓purified 15-LOX (IC_{50} = 25.0±3.0 μM) activity.	↓purified 5-LOX (IC_{50} = 2.5±0.3 μM) activity.	↓PGH synthase (IC_{50} = 20.0±2.0 μM) activity	[35]
Rat isolated peritoneal leukocytes	6-hydroxy-kaempferol 3,6-dimethyl ether; 6-hydroxy-kaempferol 3,6,4′-trimethyl ether; quercetagenin 3,6,3′-trimethyl ether; 6-hydroxy-luteolin 6-methyl ether; 6-hydroxy-luteolin 6,3′-dimethyl ether	↓TxB_2 (IC_{50} = 22 – 182 μM) and LTB_4 (IC_{50} = 58 – 182 μM) biosynthesis in A23187-stimulated cells	Not evaluated	Not evaluated	[36]
RBL-2H3	Magnolol (0.1 – 20 μM)	↓LTB_4 and LTC_4 formation (at 10 and 20 μM); no effect on IgE-induced β-hexosaminidase release; ↓AA release at 10 μM; ↓[Ca^{+2}] release in IgE-treated cells	↓LTB_4 and LTC_4 (using AA or LTA_4 as substrates) formation at 10 and	Not evaluated	[37]

			25 μM in cell-free assays		
Monocytic leukemia cells MM6, human platelets and PMNLs isolated from healthy donors.	Hyperforin (0.1 – 100 μM).	↓LTB_4 and 15-HETE formation in A23187-stimulated PMNLs (IC_{50} = 1.0-2.0 and >10 μM, respectively); ↓HHT production in thrombin- or A23187- and exogenous AA-stimulated human platelets (IC_{50} = 0.3, 3.0 and 3.0 μM, respectively) as well as in platelet lysates (IC_{50} = 3 μM).<br>Hyperforin could not interfere with COX-2 product formation (6-keto-$PGF_{1\alpha}$) in MM6 cells; and did not significantly inhibit 12-LOX or 15-LOX in intact platelets or leukocytes, respectively.	↓purified 5-LOX (IC_{50} = 90 nM) activity acting via uncompetitive mechanisms.	Not evaluated.	[38]
Rat peritoneal leukocytes	Erycristagallin (≤100 μM)	↓LTB_4 formation in A23187-stimulated leukocytes (IC_{50} = 23.4 μM) without cytotoxic effects; no effect on COX-1 from platelets at 100 μM	Not evaluated.	Not evaluated.	[39]
RAW264.7 macrophages and HT-29 human colon cancer cells.	Curcumin and THC (10, 20 and 50 μM).	↓LTB_4 and PGE_2 formation as well as AA release in LPS-stimulated RAW264.7 cells at 10 μM; ↓AA release in A23187-stimulated HT-29 cells at 10 μM; ↓$cPLA_2$ activity from microsomal fraction of HT-29 cells; ↓$cPLA_2$ level and $cPLA_2$ phosphorylation in LPS-treated RAW264.7 cells at 50 μM; ↓$PGF_{2\alpha}$, PGE_2, PGD_2, HHT, 15-HETE and 11-HETE in LPS-stimulated RAW264.7 cell lysates treated with 0THC at 50 μM; at the same concentration curcumin reduced the biosynthesis of PGD_2, 15-HETE and 11-	↓Human recombinant 5-LOX expression in LPS-treated RAW264.7 cells (IC_{50} = 0.7 and 3 μM for curcumin and THC, respectively).	↓COX-2 expression in LPS-treated RAW264.7 cells (curcumin at 20 μM), but increase COX-2 levels without LPS stimulation.	[40]

		HETE; higher inhibitory effect on the peroxidase activity of COX-1 than that of COX-2			
Rat peritoneal leukocytes.	Sigmoidin A and sigmoidin B (5 – 100 μM).	↓LTB_4 in calcium/A23187- stimulated rat peritoneal leukocytes treated with sigmoidin A (IC_{50} = 31 μM), sigmoidin B (44% inhibition at the same concentration); apigenin used as reference compound also showed inhibitory activity (IC_{50} = 14 μM). No inhibition of COX-1 activity; no reduction of 12-HHT synthesis at 100 μM of both compounds.	Not evaluated.	Not evaluated.	[41]
PMNLs from healthy donors.	Curcumin, eugenol, cinnamaldehyde and quercetin (1 – 150 μM).	↓5-HETE production in PMNLs (IC_{50} = 30.0, 26.0, 35.0 and 25.0 μM for curcumin, eugenol, cinnamaldehyde and quercetin, respectively); NDGA (IC_{50} = 28.0 μM).	Not evaluated.	Not evaluated.	[42]
Neutrophils isolated from rats	Magnolol (0.1 – 10 μM)	↓COX products formation, but low effect on 5-LOX products biosynthesis at 3 μM; Inhibition of COX and 5-LOX products production at 10 μM; ↓TxB_2 formation (IC_{50} = 0.5 μM); ↑LTB_4 biosynthesis at concentrations <3 μM, but inhibition at higher concentrations; Magnolol alone modified 5-LOX distribution in the cells, ↑AA release (PLA_2 activity), ↑$cPLA_2$ translocation and phosphorylation (effects dose-dependent) and had no effect on MAPK phosphorylation. These effects were absent in the presence of A23187.	↓COX-1 and COX-2 activity (IC_{50} = 26.0 ± 1.9 and 31.2 ± 2.8 μM, respectively) in cell-free assays	Weak inhibition of 5-LOX activity (IC_{50} ~ 90 μM) in cell-free assay	[43]

BMMC cells	Ginkgetin (1 – 50 μM).	↓LTC_4 in BMMC activated with KL (100 ng/ml) (IC_{50} = 0.33 μM); ↓PGD_2 in BMMC activated with KL (100 ng/ml), IL-10 (100 U/ml) and LPS (100 ng/ml) (IC_{50} = 0.75 μM); ↓degranulation reaction in a dose dependent manner (IC_{50} = 6.52 μM)	Not evaluated.	↓COX-2 protein level.	[44]
Rat peritoneal neutrophils and RAW 264.7 macrophages	5-*O*-demethylnobiletin (0.01 – 10 μM)	↓LTB_4 formation in rat neutrophils (IC_{50} = 0.35 μM)	↓5-LOX activity (cell lysate) by 57% at 0.25 μM	↓COX-2 expression RAW cells (5-20% inhibition)	[45]
Human monocyte line U937.	Procyanidin B_2 (50 and 100 μM).	↓LOX-1 protein and mRNA levels in ox-LDL treated macrophages.	↓5-LOX protein and mRNA levels in ox-LDL treated macrophages.	Not evaluated.	[46]
BMMC	Five coumarins (psoralen, xanthotoxin, scopoletin, umbelliferone, and (+)-marmesin) and three flavonoids (apigenin, luteolin and cynaroside) (12.5 and 25.0 μg/mL).	↓LTC_4 production in BMMC activated with KL (100 ng/ml) at 25.0 μg/mL; ↓PGD_2 formation in BMMC activated with KL (100 ng/ml), IL-10 (100 U/ml) and LPS (100 ng/ml) at 12.5 μg/mL. All compounds showed COX-2/5-LOX dual inhibitory activity.	Not evaluated.	Not evaluated.	[47]
BMMC cells	Methyl gallate (5 – 160 μM).	↓LTC_4 (IC_{50} = 5.3 μM) in BMMC activated with KL (100 ng/ml). ↓PGD_2 (IC_{50} = 17.0 μM) in BMMC activated with KL (100 ng/ml), IL-10 (100 U/ml) and LPS (100 ng/ml).	Not evaluated.	↓COX-2 activity (colorimetric COX inhibitor screening assay kit) (IC_{50} = 19.0 μM); no effect on COX-2 protein levels at ≤80 μM.	[48]
BMMC cells	Ochnaflavone (1.5 – 50 μM).	↓LTC_4 (IC_{50} = 6.5 μM) in BMMC activated with KL (100 ng/ml).	Not evaluated.	↓COX-2 protein levels.	[49]

		↓PGD_2 (IC_{50} = 0.6 μM) in BMMC activated with KL (100 ng/ml), IL-10 (100 U/ml) and LPS (100 ng/ml); ↓Degranulation reaction in a dose dependent manner (IC_{50} = 3.0 μM).			
PMNLs from healthy donors.	Eugenol (5 – 60 μM).	↓LTC_4 and 5-HETE in A23187- and AA-stimulated PMNLs (IC_{50} = 30.0 and 26.0 μM, respectively).	Not evaluated.	Not evaluated.	[50]
Human peripheral blood eosinophils	Genistein (1nM – 10 μM)	↓LTC_4 in A23187-stimulated cells (IC_{50} = 80 nM); ↓p38 and MK2 phosphorylation	↓5-LOX translocation	Not evaluated	[51]
Rat peritoneal leukocytes.	Sakuranetin, 7-*O*-methylaromadendrin and 3-acetyl-7-*O*-methylaromadendrin (5 – 100 μM).	↓LTB_4 production A23187-stimulated rat peritoneal leukocytes treated with 7-*O*-methylaromadendrin, sakuranetin, and 3-acetyl-7-*O*-methylaromadendrin (IC_{50} = 62, 9 and 15 μM, respectively); as a reference compound, apigenin also showed inhibitory effects (IC_{50} = 14 μM); ↓PLA_2 activity (only 7-*O*-methylaromadendrin); ↓Elastase release (only sakuranetin at 100 μM); none of the flavanones tested inhibited PGE_2 biosynthesis in RAW 264.7 mouse macrophages.	↓LTB_4 production in homogenized rat peritoneal polymorphonuclear leukocytes (only sakuratenin at 20 μM).	Not evaluated.	[52]
Human gastric epithelial cells (AGS) and murine macrophage RAW264.7	NDGA and geraniin (10 μM).	↓5-HETE production in AGS cells; ↓IL-8 and TNF-α in AGS cells and macrophages; attenuation of $cPLA_2$ induction by NDGA, but not geraniin.	↓5-LOX protein levels.	No effect on COX-2 protein levels.	[53]
BMMC cells	Meso-dihydroguaiaretic acid (0.8 – 25 μM).	↓LTC_4 (IC_{50} = 1.3 μM) in BMMC activated with KL (100 ng/ml); ↓PGD_2 (IC_{50} = 9.8 μM) in BMMC activated with KL (100 ng/ml), IL-10 (100 U/ml) and LPS (100 ng/ml); ↓Degranulation	Not evaluated.	No effect on COX-2 protein levels.	[54]

		reaction in a dose dependent manner (IC_{50} = 11.4 μM).			
PMNLs from healthy donors.	Carnosic acid and carnosol (0.03 – 30 μM).	↓LTB_4, 5(*S*),12(*S*)-diHETE, and 5-H(p)ETE in A23187-stimulated PMNL treated with carnosol and carnosic acid (IC_{50} = 7.0 and 15-20 μM, respectively); ↓12-HETE and 12-HPETE synthesis in A23187-stimulated PMNL treated with carnosol (IC_{50} = 13 μM), but no effect on cells treated with carnosic acid;<br>Antagonise intracellular Ca^{2+} mobilisation induced by a chemotactic stimulus, and attenuate formation of ROS and the secretion of human leukocyte elastase.	↓5-LOX activity (IC_{50} = 0.1 and 1.0 μM for carnosic acid and carnosol, respectively)	Not evaluated.	[55]
PBMC isolated from whole human blood.	Quercetin, 3′-*O*-methylquercetin (MQ), quercetin-3′-*O*-sulfate (QS), quercetin-3-*O*-glucuronide (QG) and 3′-*O*-methylquercetin-3-*O*-glucuronide (MQG) (1 – 10 μM).	↓LTB_4 synthesis in A23187-stimulated PBMCs treated with quercetin (IC_{50} = 2.0 μM) and MQ at 2.0 μM; ↓PGE_2 formation in LPS-activated PBMCs treated with quercetin and MQ (IC_{50} = 2.0 μM); lack of effect for the rest of compounds tested	Not evaluated.	Not evaluated.	[56]
Rat adherent macrophages from granuloma	NDGA (10 μM)	↓LTB_4 synthesis in stimulated cells	↓5-LOX expression (mRNA and protein)	Not evaluated	[57,58]
BBMCs isolated from mice	Isolated phenolic compounds from *Ailanthus altissima* leaves	Scopoletin, quercetin and luteolin inhibited the biosynthesis of PGD_2 (IC_{50} = 39.2, 7.3 and 2.5 μM, respectively) and LTC_4 (IC_{50} = 59.9, 5.1 and 1.8 μM, respectively); no effect of astragalin and scopoline at the concentrations tested.	Not evaluated	Not evaluated	[59]

		The methanolic extract and solvent fractions of *Ailanthus altissima* inhibited PGD_2 (49.0 – 57.4%) and LTC_4 (96.7 – 99.8%) at 50 μg/mL			
Human neutrophils	Hyperforin (0.3 – 30 μM)	↓5-LOX product biosynthesis (IC_{50} = 1.9 μM) in intact neutrophils via inhibition of 5-LOX translocation to the nuclear membrane. This effect was reduced in the presence of OAG (IC_{50} ≥ 10 μM)	↓5-LOX activity in cell homogenates (IC_{50} ~ 30 μM), in partially purified (IC_{50} ~ 7 μM) and purified enzyme (IC_{50} = 1.6 μM). This inhibition occurs via interaction with the Trp residues within the 5-LOX C2-like domain and blocking the binding of 5-LOX with CLP. Besides, this inhibitory effect was independent of the redox tone and blocked in the presence of OAG (IC_{50} ≥ 10 μM) and PC.	Not evaluated	[60]
RBL-1 cells and RAW 264.7 cells.	(-)-Nyasol (2 – 100 μM).	↓Cys-LT production in A23187-treated RBL-1 cells at 2 – 10 μM. As a reference compound, NDGA also exerted inhibitory effects at 5 μM;	Not evaluated.	No effect on COX-2 protein levels.	[61]

		↓PGD_2 and NO production in LPS-treated RAW 264.7 cells at 1 – 100 μM.			
RBL-1 and RAW 264.7 cells	*Schisandra fructus* methanolic extract and the individual compounds such as vanillic acid; NDGA as control of 5-LOX inhibition	↓5-LOX products formation (Cys-LTs) y RBL-1 cells treated with the extract (IC_{50} = 71.1 μg/mL) and NDGA (IC_{50} = 0.2 – 1 μM); vanillic acid had no effect; the extract and vanillic acid exerted less than 50% inhibition against PGE_2 and NO formation at the concentrations tested.	Not evaluated	Not evaluated	[62]
Rat endothelial cells (YPEN-1).	Morin (1 and 5 μM).	↓ROS generation; ↓NF-κB activation (via reduced DNA binding activity, IκBα phosphorylation and p65/p50 nuclear translocation); modulation of ERK and p38 signal transduction; ↓iNOS protein levels in *t*-BHP-induced YPEN-1 cells.	↓5-LOX protein levels in *t*-BHP-induced YPEN-1 cells.	↓COX-2 protein levels in *t*-BHP-induced YPEN-1 cells.	[63]
Murine macrophage RAW264.7 cells.	Curcumin (0.63 – 80 μM).	↓PGE_2 formation in LPS-stimulated RAW264.7 cells; ↑HO-1 mRNA expression and protein level; no effect on iNOS (mRNA expression and protein level), $cPLA_2$ (mRNA expression)	No effect on 5-LOX mRNA expression.	No effect on COX-2 mRNA expression and protein level.	[64]
BMCMC cells	NDGA (3 μM).	↑PGE_2 and ↓LTB_4/LTC_4 production in BMCMCs stimulated with ConA (mimics IgE cross-linking), substance P (following culture of cells with IL-4) or A23187; ↓degranulation of BMCMCs stimulated with ConA and A23187.	Not evaluated.	Not evaluated.	[65]
RBL-1	13 phenolic compounds isolated from *Lonicera japonica* (20	The compounds that exerted the highest inhibition on 5-LOX products (Cys-LTs) formation were protocatechuic acid (35.8%), 3,5-	Not evaluated	Not evaluated	[66]

	μM); NDGA as control of 5-LOX inhibition	dicaffeoylquinic acid methylester (15.1%), luteolin (97.0%), quercetin 3-O-β-D-glucopyranoside (11.1%) and NDGA (100%)			
Human isolated PBMC	Quercetin (25 μM)	↓TLR-2 and TLR-4 mRNA expression; ↓NF-κB activation; ↓IL-6 biosynthesis; ↓iNOS mRNA expression; ↓PGE_2 biosynthesis	↓5-LOX production;	↓COX-2 mRNA expression; ↓COX production;	[67]
RBL-1 cells	Scopoletin, scopolin, scoparone, esculetin chlorogenic acid, quercetin, isorhamnetin-3-*O*-galactoside, isorhamnetin-3-*O*-robinobioside, capillarisin, and NDGA (0.1 – 50 μM)	↓LTB_4 biosynthesis in A23187-stimulated cells. The most effective compounds were NDGA (95.7% inhibition at 1 μM), as well as esculetin, quercetin, and capillarisin (IC_{50} = 6.6, 0.7 and 17.7 μM, respectively).	Not evaluated	Not evaluated	[68]
Rat peritoneal macrophages	*Platycladus orientalis* chloroform extract (12.5 – 100 μg/mL) and the individual compounds hinokiol and acacetin (12.5 – 100 μM); 12.5 μM NDGA was used as control of 5-LOX inhibition	↓5-HETE and LTB_4; ↓12-HHT; the extract showed no inhibitory effect on LTA_4 hydrolase	↓5-LOX activity (43.2% for honokiol and 48.8% for acacetin at 100 μM; IC_{50} = 7.1 μM for NDGA) in a cell-free assay	Moderate or weak COX-2 inhibition in cell-free assays	[69]
RBL-2H3 cells	Extracts of *Foeniculum vulgare* (12.5 – 200 mg/mL), its individual compounds (50 μM) and 1 μM NDGA as positive control	↓5-LOX products (Cys-LTs) formation in cells treated with the extracts (only at 200 μg/mL); the individual phenolics (scopoletin and umbelliferone) had no effect on 5-LOX activity; 1μM NDGA exerted a strong inhibition (92.4%)	Not evaluated	Not evaluated	[70]

RBL-2H3 cells	Macelignan (5, 10 and 20 μM)	No cytotoxic effect; ↓β-hexosaminidase (8 – 32% inhibition) and histamine release (8 – 27% inhibition); ↓Ca^{+2} intracellular concentration; ↓PGE_2 and LTC_4 biosynthesis; ↓IL-4, IL-13, and TNF-α formation; ↓Akt and MAPK phosphorylation	↓5-LOX mRNA expression	↓COX-2 mRNA expression	[71]
PMNL from healthy donors, whole human blood and HEK293 cells	Caffeic acid and CAPE (0.1-30 μM).	CAPE exerted a ↓LT biosynthesis (LTB_4, 20-COOH- and 20-OH-LTB_4 as well as 5-HETE) in thapsigargin-stimulated PMNLs (53-85% inhibition at 1 μM; IC_{50} = 0.52 μM) as well as in zymosan-stimulated whole blood treated (32% reduction at 1 μM; IC_{50} = 1.79 μM); CAPE also exerted a ↓AA release in thapsigargin-stimulated PMNLs (32% reduction at 1 μM); Caffeic acid exerted no effects.	CAPE exerted ↓5-LOX activity (IC_{50} = 0.13 μM) whereas caffeic acid had no effect on the enzyme isolated from 5-LOX-transfected HEK293	Not evaluated.	[72]
Rat basophilic leukemia (RBL-2H3) cells.	Panduratin A (1 – 20 μM).	The effects observed in A23187- and PMA-stimulated RBL-2H3 cells were: ↓LTB_4 and PGE_2 formation as well as ↓AA release; ↓Ca^{2+} influx and β-hexosaminidase and histamine secretion at 20 μM; ↓IL-4, IL-13, and TNF-α mRNA expression; ↓Akt, ERK, p38, and JNK phosphorylation	↓5-LOX mRNA expression.	↓COX-2 mRNA expression.	[73]
Primary avian polymorphonuclear leukocytes.	Caffeic acid (5 μM).	↓LTB_4 production in leukocytes stimulated with TLR agonists (FLG, PAM or CpG).	Not evaluated.	Not evaluated.	[74]
Feline esophageal epithelial cells.	Eupatilin (25-200 μM).	↓H_2O_2-induced cytotoxicity (dose-dependent effect), ↓p38 and JNK activation, and LTB_4 production at 150 μM (12 h pretreatment).	↓5-LOX expression in H_2O_2-induced cells at 150 μM (12 h pre-treatment)	Not evaluated.	[75]

Human neutrophils isolated from peripheral blood of healthy donors.	3,3′,4′-Tri-hydroxy-flavone, 3,5,7-tri-hydroxy-flavone, 3,7,3′-tri-hydroxy-flavone and 3,7,4′-tri-hydroxy-flavone (10 and 25 μM), NDGA and quercetin (10 μM)	↓LTB_4 production in A23187- and AA-stimulated neutrophils (except for 3,7,3′-tri-hydroxy-flavone at 10 μM); ↓oxidation of luminol, amplex red and APF by reactive species generated by PMA-stimulated neutrophils; ↓COX-1 activity in a cell-free system for 3,5,7-tri-hydroxy-flavone and 3,7,3′-tri-hydroxy-flavone in a concentration-dependent manner.	Not evaluated.	↓COX-2 activity in a cell-free system for 3,5,7-tri-hydroxyflavone in a concentration-dependent manner.	[76]
RAW264.7 cells and rat peritoneal macrophages.	Baicalein and its glycoside, baicalin (1.5 – 100 μM).	↓LTB_4 production in rat peritoneal macrophages in the presence of exogenous AA (IC_{50} = 35.6 μM); ↓PGE_2 in LPS-stimulated RAW264.7 cells by baicalein (IC_{50} = 28.6 μM).	No effect on 5-LOX expression (mRNA and protein level).	↓COX-2 mRNA expression and protein level in LPS-stimulated RAW264.7 cells.	[77]
Human isolated PMNL or monocytes	Cannflavin A and B (0.1 – 10 μM)	↓5-LOX products (LTB_4, tr-LTB_4, 5-HPETE) with IC_{50} = 1.6 – 2.4 μM for cannaflavin A; ↓PGE_2 biosynthesis (IC_{50} = 8.8 μM for cannaflavin A) in LPS stimulated monocytes; ↓12-HHT in platelets (IC_{50} > 10 μM for cannaflavin A)	↓5-LOX (IC_{50} = 0.8 and 0.9 μM for cannaflavin A and B, respectively) activity	↓mPGES-1 activity by both compounds (cannaflavin A showed IC_{50} = 1.8 μM); ↓COX-1 and COX-2 activity (cannaflavin A showed IC_{50} > 10 μM)	[78]
Human isolated neutrophils	Luteolin, NDGA, quercetin, 3′,4′-dihydroxyflavone, apigenin, acacetin, chrysoeriol, diosmetin, taxifolin, chrysin, (±)-eriodictyol (±)-naringenin and other related flavonoids (10 and 40 μM)	The highest inhibition of LTB_4 synthesis was exerted by luteolin (IC_{50} = 1.6 ± 0.3 μM), 3′,4′-dihydroxyflavone (IC_{50} = 1.7 ± 0.1 μM), quercetin (IC_{50} = 4.0 ± 1.2 μM), NDGA (56.5 ± 2.5% inhibition at 1μM) and eriodyctiol (53.4 ± 4.8% inhibition at 40 μM).	Not evaluated	Not evaluated	[79]

BMMC	Homoisoflavanone (5,7-di-hydroxy-3-(3-hydroxy-4-methoxy-benzyl)-chroman-4-one) (1.4 – 14.4 μM).	↓LTB_4, LTC_4 and PGD_2 in DNP-IgE and DNP-HSA-activated BMMCs; ↓mRNA level and release of IL-6 and TNF-α; ↓degranulation of BMCMCs; no effect on COX-1 protein levels	↓5-LOX protein levels in DNP-IgE and DNP-HSA-activated BMMCs.	↓COX-2 protein levels in DNP-IgE and DNP-HSA-activated BMMCs.	[80]
BMMCs-isolated from bone marrow of C57BL/6	Curcumin (1 – 10 μM)	↓LTC_4 and Ca^{+2} influx in IgE/Ag-induced BMMCs; ↓COX-2-dependent PGD_2 synthesis; ↓$cPLA_2$ phosphorylation and translocation; inhibition of NF-κB, MAPK, and Syk pathways (all the effects were dose-dependent).	↓5-LOX translocation	Not evaluated	[81]
PMNL from healthy donors.	Pinostilbene, rhapontigenin, isorhapontigenin, RSV, oxyresveratrol, pterostilbene	↓LTB_4 (IC_{50} = 18.5 ± 7.8 and 9.32 ± 3.3 μM for oxyresveratrol and pterostilbene, respectively; IC_{50} > 50 μM for the rest of compounds) Pinostilbene exerted the highest COX-1 and COX-2 inhibition (IC_{50} = 1.9 ± 1.6 and 0.35 ± 0.23, respectively), whereas rhapontigenin was the weakest compound (IC_{50} = 24.55 ± 10.38 and 36.12 ± 10.43, respectively)	Molecular docking analysis showed that pterostilbene formed hydrogen bonds with His372 and Thr364	Molecular docking analysis showed that pinostilbene formed hydrogen bonds with Arg129, Phe518 and Gln192;	[82]
PMNL from healthy donors.	Xanthohumol, xanthohumol C, 8-prenylnaringenin, 4-hydroxy-colupulone, humulone, cascadone and humudifucol (up to 10 μM).	↓LTB_4 and 5-HPETE in A23187- and AA-stimulated PMNLs treated with xanthohumol (IC_{50} = 2.9 μM); no effects observed with the rest of compounds.	Not evaluated.	Not evaluated.	[83]
Human peripheral neutrophils from human whole blood.	Daidzein, dihydrodaidzein, and equol (0.01 – 1 μM).	↓LTB_4 A23187- and AA- stimulated neutrophils (IC_{50} = 0.2 μM for equol; 20% of inhibition for daidzein and dihydrodaidzein 1 μM); no effect on the enzymatic hydrolysis of LTA_4 to LTB_4; ↓free radical peroxidation of AA (IC_{50}	Not evaluated.	Not evaluated.	[84]

		= 0.6 μM for daidzein and >1.0 μM for equol and dihydrodaidzein); ↓MPO activity (IC_{50} = 0.45, 0.7 and >1.0 μM for equol, dihydrodaidzein and daidzein, respectively)			
Isolated human PMNL	CAPE (1 μM)	↓5-LOX products	Molecular docking analysis showed affinity = -8.5 Kcal/mol and interaction with His372	Not evaluated.	[85]
RAW 264.7 macrophages	Isoorientin (1 – 25 μM; 16 h)	↓TNF-α, IL-1β, iNOS expression and NF-κB pathway;	↓5-LOX protein expression	↓COX-2 protein expression	[86]
Mouse mast cell line (PB-3c).	Daidzein, genistein and their glycosides (daidzin and genistin), equol, quercetin and kaempferol (50 μM; 48 h).	↓LTB_4 production A23187- and AA-stimulated PB-3c cells (100% inhibition exerted by equol, quercetin and kaempferol, 72.5% by genistein, and a weak but significant suppression by daidzin and daidzein).	Not evaluated.	Not evaluated.	[87]
Isolated human PMNL and platelets	Baicalein, apigenin, quercetin, CAPE, myricetin (0.1 – 20 μM)	↓5-LOX products (including LTB_4, 6-*trans*-LTB_4, 6-*trans*-12-*epi*-LTB_4, 20-OH-LTB_4, 20-COOH-LTB_4, and 5-HETE); IC_{50} = 11.0 and 5.97 μM in PMNL for baicalein and quecetin, respectively). ↓12-HETE (IC_{50} = 1.78, 1.34 and 1.28 μM, respectively) and 12-HHT (IC_{50} = 9.92, 2.91 and 3.79 μM, respectively) in platelets treated with baicalein, quercetin and CAPE	Molecular docking analysis using soybean 15-LOX; (i) CAPE´s affinity = -5.4 kcal/mol and interaction with Arg426, Ser510, His518 and Phe576 (ii) Baicalein´s affinity = -1.4 kcal/mol and interaction with	Not evaluated.	[88]

			Fe, His518 and Phe576<br>(iii) Quercetin´s affinity = 0.8 kcal/mol and interaction with Asp766, His518 and Phe576		
Isolated human PMNL and whole blood	CAPE (0.1 – 10 μM)	↓5-LOX products (including LTB_4, tr-LTB_4, 20-OH-LTB_4, 20-COOH-LTB_4, and 5-HETE); IC_{50} = 0.97 and 3.58 μM in PMNL and whole blood, respectively)	Molecular docking analysis into the active site of 5-LOX (cristal structure) showed affinity = -8.8 Kcal/mol and interaction with Leu420 and His372	Not evaluated	[89]
Caco-2	Hydroxytyrosol (1 μM)	↓PGE_2, LTB_4, 5-HETE, 12-HETE, 15-HETE, 13-HODE	Not evaluated.	Not evaluated.	[90]
RAW 264.7 macrophages	Ellagic acid-3,3′,4-trimethoxy-4′-*O*-α-L-rhamnopyranoside (1 – 100 μM for toxicity assays; 1 – 5 μM for anti-inflammatory assays)	↓NO, TNF-α, IL-6 and NF-κB expression; ↑IL-10	↓5-LOX level in culture medium	↓COX-2 level in culture medium	[91]
Human monocytes, neutrophils and platelets.	Ginkgolic acid (0.1 – 10 μM).	↓Tr-LTB_4 isomers and 5-HPETE in A23187-stimulated intact human neutrophils in the presence or absence of exogenous AA (IC_{50} = 3.8 and 2.9 μM, respectively); ↓12-HHT formation by isolated COX-1 (IC_{50} = 8.1 μM); ↓12-HHT and TxB_2 formation in AA-stimulated platelets (IC_{50} = 2.1 and 2.2 μM,	↓Tr-LTB_4 isomers and 5-HPETE synthesized by human recombinant 5-LOX (IC_{50} = 0.2 μM)	No effect on COX-2 activity (human recombinant isolated enzyme)	[92]

		respectively); ↓PGE_2, PGD_2, TxB_2, $PGF_{2\alpha}$, 11-HETE, 12-HHT, PGE_1 and TxB_1 in LPS-stimulated monocytes.			
PBMCs from healthy donors.	Apigenin, EGCG, genistein, naringenin, nobiletin, wogonin), RSV and its dimer (ε-viniferin), tetramer (hopeaphenol), and imine analogues (IRA) (1 – 10 μM)	↓LTB_4 production by A23187- stimulated neutrophils treated with genistein, resveratrol, IRA (1 and 10 μM) and ε-viniferin (10 μM); LC-MS based targeted oxylipin metabolomics showed that: RSV inhibited the COX-1/2 pathway and exerted a weak attenuation of the 12/15-LOX activity; ε-viniferin caused a clear substrate shunt towards the remaining AA cascade enzymes (15-LOX, COX-1/2, cytochrome P450); IRA had no impact on 15-LOX activity, but elevated the formation of COX-derived PGs, having no inhibitory effects on COX-1/2 activity.	Not evaluated.	Not evaluated.	[93]
Murine mast cell line (MC/9).	Kuwanon G and Morusin (0.5–10 μM).	↓LTB_4 production in PMA- and A23187-stimulated MC/9 mast cells treated with morusin (2 and 4 μM); ↓histamine production by kuwanon G (2.5–10 μM) and morusin (4 μM).	↓5-LOX nuclear protein level, but not cytoplasmatic level in PMA- and A23187- stimulated MC/9 mast cells treated with kuwanon G (2.5 – 10 μM) and morusin (4 μM)	Not evaluated.	[94]
BMMC	Taxifolin (dihydroquercetin) (1, 2 and 5 μM).	↓LTC_4 in IgE/Ag-stimulated and DNP-HSA-activated BMMCs.	↓5-LOX nuclear protein levels in	↓COX-2 protein levels in IgE/Ag-stimulated and	[95]

		↓IL-6 and MAPKs protein levels, translocation of $cPLA_2$ and Akt/IKK/NF-κB pathway in IgE/Ag-stimulated and DNP-HSA-activated BMMCs. ↓degranulation, histamine release, phosphorylation of PLCγ, and Ca^{2+} mobilization in IgE/Ag-stimulated and DNP-HSA-activated BMMCs.	IgE/Ag-stimulated and DNP-HSA-activated BMMCs.	DNP-HSA-activated BMMCs.	
PMNLs from healthy donors.	Uro-A, Uro-B, Uro-C and IsoUro-A, and their conjugates Uro-A glur, Uro-B glur, IsoUro-A-glur and Uro-A sulfate (1, 5 and 15 μM).	↓LTB_4 and 5-HETE (dose-dependent) synthesis in A23187/LPS-stimulated PMNLs treated with Uro-C (15 μM); ↓PGE_2 formation A23187/LPS- stimulated PMNLs treated with Uro-A or IsoUro-A (15 μM); ↓HKE_2 and HKD_2 production (5-LOX/COX-2 pathway) in A23187/LPS-stimulated PMNLs treated with Uro-A, IsoUro-A, and Uro-C at 15 μM; ↓HKD_2 in the cells treated with Uro-B at 15 μM.	↑No effect on 5-LOX protein level in A23187/LPS-stimulated PMNLs treated with Uro-C at 15 μM.	↓COX-2 protein level in A23187/LPS-stimulated PMNLs treated with Uro-A and IsoUro-A at 15 μM; no effect of these compounds on COX-2 activity	[96]
Caco-2	5,7 di-hydroxy-3,3′,4′-trimethoxyflavone and 3,5,6,7,4′-pentamethoxy-flavone	↓TNF-α and IL-1β as well as NF-κB activation compared to LPS-treated cells.	No effect on 5-LOX expression	↓COX-2 expression in LPS-treated cells	[97]
Human neutrophils	NDGA (1 μM)	↓TLB_4, tr-LTB_4, 20-OH-LTB_4, 5-HETE, 12-HETE and 15-HETE	No effect on 5-LOX expression	No effect on COX-2 expression	[98]
RAW 264.7 macrophages	Celery ethanol extract, and karafsin (apigenin-7-O-β-(5′-E-p-coumaroyl)-β-apiofuranoside)	No cytotoxic effects; ↓NO release (0.0625 – 1 mg/mL).	↓5-LOX activity (25 – 500 mg/mL)	↓COX-2 activity (25 – 500 mg/mL)	[99]

Rat basophilic leukemia cell line (RBL-2H3) cells.	Tricin (30.2 μM – 1.5 mM).	↓LTB_4, LTC_4 and PGE_2 formation in anti-DNP-IgE plus DNP-HAS-stimulated RBL-2H3 cells; ↓hexosaminidase release; ↓IL-4 and TNF-α level; ↓cPLA2, Akt, ERK, p38, JNK, protein kinase C, phospholipase C1, Lyn and Syk phosphorylation; minimal effect on Fyn.	↓5-LOX protein levels in anti-DNP-IgE plus DNP-HAS-stimulated RBL-2H3 cells.	↓COX-2 protein levels in anti-DNP-IgE plus DNP-HAS-stimulated RBL-2H3 cells.	[100]
Rat basophilic leukemia RBL-2H3 cells and human non–small-cell lung carcinoma A549 cells.	Red-kerneled rice proanthocyanidin -RRP- (catechin octamer) and catechin monomer (1 – 10 μM).	↓LTB_4 and PGE_2 formation in A23187-stimulated RBL-2H3 and A549 cells treated with RRP at 1 μM;<br>RRP exerted inhibition of 12-LOX (IC_{50} = 39.0 μM) and p12-LOX (IC_{50} = 47.5 μM) activity; weak inhibitory effect on 15-LOX-2 (IC_{50} >100 μM); catechin exerted ↓15-LOX-2 (IC_{50} = 20.4 μM) and COX-1 (IC_{50} = 30 μM)	RRP and catechin exerted non-competitive inhibition against human 5-LOX (IC_{50} = 15.1 and 2.2 μM, respectively) and rat 5-LOX (IC_{50} = 7.0 and 9.0 μM, respectively)	RRP exerted no effect on COX-1 or COX-2 activity up to 500 μM	[101]

Abbreviations: **AA**, arachidonic acid; **ADP**, adenosine diphosphate; **APF**, 2-[6-(4′-amino)phenoxy-3H-xanthen-3-on-9-yl]benzoic acid; **BMCMCs**, canine bone marrow derived cultured mast cells; **BMMC**, mice bone marrow-derived mast cells; **cAMP**, cyclic adenosine monophosphate; **CAPE**, caffeic acid phenethyl ester; **CLP**, coactosin-like protein; **ConA**, concanavalin A; **COX**, cyclooxygenase; **CpG**, unmethylated CpG motifs of bacteria DNA; **cPLA2**, cytoplasmic phospholipase-2; **Cys-LTs**, cysteinyl leukotrienes; **DNP-HAS**, dinitrophenyl human serum albumin; **DNP-IgE**, dinitrophenyl immunoglobulin E; **DPE**, 2-(3,4-dihydroxy-phenyl)-ethanol; **EGCG**, epigallocatechin gallate; **FLG**, flagellin; **fMLP**, formyl methionyl leucyl phenylalamine; **HETE**, hydroxyeicosatetraenoic acid; **HHT**, hydroxy-5,8,10-heptadecatetraenoic acid; **HKE$_2$**, hemiketal E_2; **HKD$_2$**, hemiketal D_2; **HODE**, hydroxyoctadecadienoic acid; **HO-1**, hemeoxygenase-1; **HPETE**, hydroxyperoxyeicosatetraenoic acid; **iNOS**, inducible nitric oxide synthase; **KL**, c-kit ligand; **LOX**, lipoxygenase; **LOX-1**, lectin-like oxidized low-density lipoprotein receptor-1; **LPS**, lipopolysaccharide; **LT**, leukotrienes; **MAPK**, mitogen activated protein kinase; **MK2**, mitogen-activated protein kinase-activated protein kinase2; **mPGES**, microsomal prostaglandin E synthase; **MPO**, myeloperoxidase; **MQ**, 3′-*O*-methylquercetin; **MQG**, 3′-*O*-methylquercetin-3-*O*-glucuronide; **Myr-3-glur**; myricetin-3-*O*-β-D-glucuronide; **NDGA**, nordihydroguaiaretic acid; **NF-κB**, nuclear factor kappa-light-chain-enhancer of activated B cells; **NO**, nitric oxide; **OAG**, 1-oleoyl-2-acetylglycerol; **ox-LDL**, oxidized low density lipoprotein; **p12-LOX**, porcine leukocyte-like 12 lipoxygenase; **PAM**, bacterial lipoprotein mimic palmitoly-3-cysteine–serine–lysine-4; **PBMCs**, peripheral blood mononuclear cells; **PC**, phosphatidil coline; **PG**, prostaglandins; **PLA$_2$**, phospholipase-2; **PMA**, phorbol 12-myristate 13-acetate; **PMNLs**, polymorphonuclear leukocytes; **QG**, quercetin-3-*O*-glucuronide; **QS**, quercetin-3′-*O*-sulfate; **ROS**, reactive oxygen species; **RRP**, red-kerneled rice proanthocyanidin; **RSV**, resveratrol; **THC**, tetrahydrocurcumin; **TLR**, toll-like receptor; **TNF-α**, tumor necrosis factor alpha; **Tr-LTB$_4$**, trans-LTB_4; **Trp**, tryptophan; **TxB$_2$**, thromboxane B_2; **Uro**, urolithins;

References

1. Bokoch, G.M.; Reed, P.W. Evidence for Inhibition of Leukotriene A4 Synthesis by 5,8,11,14-Eicosatetraynoic Acid in Guinea Pig Polymorphonuclear Leukocytes. *J. Biol. Chem.* **1981**, *256*, 4156–4159.
2. Chang, J.; Skowronek, M.D.; Cherney, M.L.; Lewis, A.J. Differential Effects of Putative Lipoxygenase Inhibitors on Arachidonic Acid Metabolism in Cell-Free and Intact Cell Preparations. *Inflammation* **1984**, *8*, 143–155, doi:10.1007/BF00916090.
3. Koshihara, Y.; Neichi, T.; Murota, S.; Lao, A.; Fujimoto, Y.; Tatsuno, T. Caffeic Acid Is a Selective Inhibitor for Leukotriene Biosynthesis. *Biochim. Biophys. Acta* **1984**, *792*, 92–97.
4. Kuhl, P.; Shiloh, P.; Jha, H.; Murawski, U.; Zilliken, F. 6,7,4′-Trihydroxyisoflavan: A Potent and Selective Inhibitor of 5-Lipoxygenase in Human and Porcine Peripheral Blood Leukocytes. *Prostaglandins* **1984**, *28*, 783–804, doi:10.1016/0090-6980(84)90035-2.
5. Salari, H.; Braquet, P.; Borgeat, P. Comparative Effects of Indomethacin, Acetylenic Acids, 15-HETE, Nordihydroguaiaretic Acid and BW755C on the Metabolism of Arachidonic Acid in Human Leukocytes and Platelets. *Prostaglandins. Leukot. Med.* **1984**, *13*, 53–60, doi:10.1016/0262-1746(84)90102-1.
6. Sirois, P.; Saura, C.; Salari, H.; Borgeat, P. Comparative Effects of Etodolac, Indomethacin, and Benoxaprofen on Icosanoid Biosynthesis. *Inflammation* **1984**, *8*, 353–364, doi:10.1007/BF00918212.
7. Bremm, K.D.; König, W.; Pfeiffer, P.; Rauschen, I.; Theobald, K.; Thelestam, M.; Alouf, J.E. Effect of Thiol-Activated Toxins (Streptolysin O, Alveolysin, and Theta Toxin) on the Generation of Leukotrienes and Leukotriene-Inducing and -Metabolizing Enzymes from Human Polymorphonuclear Granulocytes. *Infect. Immun.* **1985**, *50*, 844–851, doi:10.1128/iai.50.3.844-851.1985.
8. Reiner, N.E.; Malemud, C.J. Arachidonic Acid Metabolism by Murine Peritoneal Macrophages Infected with Leishmania Donovani: In Vitro Evidence for Parasite-Induced Alterations in Cyclooxygenase and Lipoxygenase Pathways. *J. Immunol. Baltim. Md 1950* **1985**, *134*, 556–563.
9. Boctor, A.M.; Pugsley, T.A. Effect of CI-922, a Potential New Antiallergy Agent, on Arachidonic Acid Metabolism in Vitro. *Inflammation* **1986**, *10*, 435–441, doi:10.1007/BF00915827.
10. Dreyling, K.W.; Hoppe, U.; Peskar, B.A.; Morgenroth, K.; Kozuschek, W.; Peskar, B.M. Leukotriene Synthesis by Human Gastrointestinal Tissues. *Biochim. Biophys. Acta* **1986**, *878*, 184–193, doi:10.1016/0005-2760(86)90145-1.
11. Nielsen, O.H.; Bukhave, K.; Ahnfelt-Rønne, I.; Elmgreen, J. Arachidonic Acid Metabolism in Human Neutrophils: Lack of Effect of Cyclosporine A. *Int. J. Immunopharmacol.* **1986**, *8*, 419–426, doi:10.1016/0192-0561(86)90126-8.
12. Flynn, D.L.; Rafferty, M.F.; Boctor, A.M. Inhibition of Human Neutrophil 5-Lipoxygenase Activity by Gingerdione, Shogaol, Capsaicin and Related Pungent Compounds. *Prostaglandins. Leukot. Med.* **1986**, *24*, 195–198, doi:10.1016/0262-1746(86)90126-5.

13. Kimura, Y.; Okuda, H.; Okuda, T.; Hatano, T.; Arichi, S. Studies on the Activities of Tannins and Related Compounds, X. Effects of Caffeetannins and Related Compounds on Arachidonate Metabolism in Human Polymorphonuclear Leukocytes. *J. Nat. Prod.* **1987**, *50*, 392–399, doi:10.1021/np50051a009.
14. Moroney, M.A.; Alcaraz, M.J.; Forder, R.A.; Carey, F.; Hoult, J.R. Selectivity of Neutrophil 5-Lipoxygenase and Cyclo-Oxygenase Inhibition by an Anti-Inflammatory Flavonoid Glycoside and Related Aglycone Flavonoids. *J. Pharm. Pharmacol.* **1988**, *40*, 787–792, doi:10.1111/j.2042-7158.1988.tb05173.x.
15. Laughton, M.J.; Evans, P.J.; Moroney, M.A.; Hoult, J.R.; Halliwell, B. Inhibition of Mammalian 5-Lipoxygenase and Cyclo-Oxygenase by Flavonoids and Phenolic Dietary Additives. Relationship to Antioxidant Activity and to Iron Ion-Reducing Ability. *Biochem. Pharmacol.* **1991**, *42*, 1673–1681, doi:10.1016/0006-2952(91)90501-u.
16. Yamamoto, S.; Aizu, E.; Jiang, H.; Nakadate, T.; Kiyoto, I.; Wang, J.C.; Kato, R. The Potent Anti-Tumor-Promoting Agent Isoliquiritigenin. *Carcinogenesis* **1991**, *12*, 317–323, doi:10.1093/carcin/12.2.317.
17. Voss, C.; Sepulveda-Boza, S.; Zilliken, F.W. New Isoflavonoids as Inhibitors of Porcine 5-Lipoxygenase. *Biochem. Pharmacol.* **1992**, *44*, 157–162, doi:10.1016/0006-2952(92)90049-o.
18. Ammon, H.P.; Safayhi, H.; Mack, T.; Sabieraj, J. Mechanism of Antiinflammatory Actions of Curcumine and Boswellic Acids. *J. Ethnopharmacol.* **1993**, *38*, 113–119, doi:10.1016/0378-8741(93)90005-p.
19. Butenko, I.G.; Gladtchenko, S.V.; Galushko, S.V. Anti-Inflammatory Properties and Inhibition of Leukotriene C4 Biosynthesis in Vitro by Flavonoid Baicalein from Scutellaria Baicalensis Georgy Roots. *Agents Actions* **1993**, *39 Spec No*, C49-51, doi:10.1007/BF01972717.
20. Glaser, K.B.; Sung, A.; Bauer, J.; Weichman, B.M. Regulation of Eicosanoid Biosynthesis in the Macrophage. Involvement of Protein Tyrosine Phosphorylation and Modulation by Selective Protein Tyrosine Kinase Inhibitors. *Biochem. Pharmacol.* **1993**, *45*, 711–721, doi:10.1016/0006-2952(93)90147-o.
21. HOULT, J.R.S.; PANG, L. -H.; BLAND-WARD, P.A.; FORDER, R.A.; WILLIAMS, C.A.; HARBORNE, J.B. Inhibition of Leucocyte 5-Lipoxygenase and Cyclo-oxygenase but Not Constitutive Nitric Oxide Synthase by Tanetin, a Novel Flavonol Derived from Feverfew, Tanacetum Parthenium. *Pharm. Pharmacol. Commun.* **1995**, *1*, 71–74, doi:10.1111/j.2042-7158.1995.tb00394.x.
22. Kimura, Y.; Okuda, H.; Kubo, M. Effects of Stilbenes Isolated from Medicinal Plants on Arachidonate Metabolism and Degranulation in Human Polymorphonuclear Leukocytes. *J. Ethnopharmacol.* **1995**, *45*, 131–139, doi:10.1016/0378-8741(94)01206-f.
23. Hamasaki, Y.; Muro, E.; Miyanji, S.; Yamamoto, S.; Kobayashi, I.; Sato, R.; Zaitu, M.; Matsuo, M.; Ichimaru, T.; Tasaki, H.; et al. Inhibition of Leukotriene Synthesis by Honokiol in Rat Basophilic Leukemia Cells. *Int. Arch. Allergy Immunol.* **1996**, *110*, 278–281, doi:10.1159/000237299.
24. Dehmlow, C.; Erhard, J.; de Groot, H. Inhibition of Kupffer Cell Functions as an Explanation for the Hepatoprotective Properties of Silibinin. *Hepatol. Baltim. Md* **1996**, *23*, 749–754, doi:10.1053/jhep.1996.v23.pm0008666328.
25. Dehmlow, C.; Murawski, N.; de Groot, H. Scavenging of Reactive Oxygen Species and Inhibition of Arachidonic Acid Metabolism by Silibinin in Human Cells. *Life Sci.* **1996**, *58*, 1591–1600, doi:10.1016/0024-3205(96)00134-8.

26. Kohyama, N.; Nagata, T.; Fujimoto, S.; Sekiya, K. Inhibition of Arachidonate Lipoxygenase Activities by 2-(3,4-Dihydroxyphenyl)Ethanol, a Phenolic Compound from Olives. *Biosci. Biotechnol. Biochem.* **1997**, *61*, 347–350, doi:10.1271/bbb.61.347.
27. Petroni, A.; Blasevich, M.; Papini, N.; Salami, M.; Sala, A.; Galli, C. Inhibition of Leukocyte Leukotriene B4 Production by an Olive Oil-Derived Phenol Identified by Mass-Spectrometry. *Thromb. Res.* **1997**, *87*, 315–322, doi:10.1016/s0049-3848(97)00133-3.
28. Yamazaki, R.; Aiyama, R.; Matsuzaki, T.; Hashimoto, S.; Yokokura, T. Anti-Inflammatory Effect of YPE-01, a Novel Diarylheptanoid Derivative, on Dermal Inflammation in Mice. *Inflamm. Res. Off. J. Eur. Histamine Res. Soc. Al* **1998**, *47*, 182–186, doi:10.1007/s000110050315.
29. Rotondo, S.; Rajtar, G.; Manarini, S.; Celardo, A.; Rotillo, D.; de Gaetano, G.; Evangelista, V.; Cerletti, C. Effect of Trans-Resveratrol, a Natural Polyphenolic Compound, on Human Polymorphonuclear Leukocyte Function. *Br. J. Pharmacol.* **1998**, *123*, 1691–1699, doi:10.1038/sj.bjp.0701784.
30. Müller, K.; Ziereis, K.; Paper, D.H. Ilex Aquifolium: Protection against Enzymatic and Non-Enzymatic Lipid Peroxidation. *Planta Med.* **1998**, *64*, 536–540, doi:10.1055/s-2006-957509.
31. Hiermann, A.; Schramm, H.W.; Laufer, S. Anti-Inflammatory Activity of Myricetin-3-O-b-D-Glucuronide and Related Compounds. **1998**, *47*, 7.
32. de la Puerta, R.; Forder, R.A.; Hoult, J.R. Inhibition of Leukocyte Eicosanoid Generation and Radical Scavenging Activity by Gnaphalin, a Lipophilic Flavonol Isolated from Helichrysum Picardii. *Planta Med.* **1999**, *65*, 507–511, doi:10.1055/s-1999-14005.
33. de la Puerta, R.; Ruiz Gutierrez, V.; Hoult, J.R. Inhibition of Leukocyte 5-Lipoxygenase by Phenolics from Virgin Olive Oil. *Biochem. Pharmacol.* **1999**, *57*, 445–449, doi:10.1016/s0006-2952(98)00320-7.
34. Alanko, J.; Riutta, A.; Holm, P.; Mucha, I.; Vapaatalo, H.; Metsä-Ketelä, T. Modulation of Arachidonic Acid Metabolism by Phenols: Relation to Their Structure and Antioxidant/Prooxidant Properties. *Free Radic. Biol. Med.* **1999**, *26*, 193–201, doi:10.1016/s0891-5849(98)00179-8.
35. MacCarrone, M.; Lorenzon, T.; Guerrieri, P.; Agrò, A.F. Resveratrol Prevents Apoptosis in K562 Cells by Inhibiting Lipoxygenase and Cyclooxygenase Activity. *Eur. J. Biochem.* **1999**, *265*, 27–34, doi:10.1046/j.1432-1327.1999.00630.x.
36. Williams, C.A.; Harborne, J.B.; Geiger, H.; Hoult, J.R. The Flavonoids of Tanacetum Parthenium and T. Vulgare and Their Anti-Inflammatory Properties. *Phytochemistry* **1999**, *51*, 417–423, doi:10.1016/s0031-9422(99)00021-7.
37. Hamasaki, Y.; Kobayashi, I.; Zaitu, M.; Tsuji, K.; Kita, M.; Hayasaki, R.; Muro, E.; Yamamoto, S.; Matsuo, M.; Ichimaru, T.; et al. Magnolol Inhibits Leukotriene Synthesis in Rat Basophilic Leukemia-2H3 Cells. *Planta Med.* **1999**, *65*, 222–226, doi:10.1055/s-1999-13984.
38. Albert, D.; Zündorf, I.; Dingermann, T.; Müller, W.E.; Steinhilber, D.; Werz, O. Hyperforin Is a Dual Inhibitor of Cyclooxygenase-1 and 5-Lipoxygenase. *Biochem. Pharmacol.* **2002**, *64*, 1767–1775, doi:10.1016/s0006-2952(02)01387-4.
39. Njamen, D. Anti-Inflammatory Activity of Erycristagallin, a Pterocarpene from Erythrina Mildbraedii. *Eur. J. Pharmacol.* **2003**, *468*, 67–74, doi:10.1016/S0014-2999(03)01664-9.

40. Hong, J.; Bose, M.; Ju, J.; Ryu, J.-H.; Chen, X.; Sang, S.; Lee, M.-J.; Yang, C.S. Modulation of Arachidonic Acid Metabolism by Curcumin and Related Beta-Diketone Derivatives: Effects on Cytosolic Phospholipase A(2), Cyclooxygenases and 5-Lipoxygenase. *Carcinogenesis* **2004**, *25*, 1671–1679, doi:10.1093/carcin/bgh165.
41. Njamen, D.; Mbafor, J.T.; Fomum, Z.T.; Kamanyi, A.; Mbanya, J.-C.; Recio, M.C.; Giner, R.M.; Máñez, S.; Ríos, J.L. Anti-Inflammatory Activities of Two Flavanones, Sigmoidin A and Sigmoidin B, from Erythrina Sigmoidea. *Planta Med.* **2004**, *70*, 104–107, doi:10.1055/s-2004-815484.
42. Prasad, N.S.; Raghavendra, R.; Lokesh, B.R.; Naidu, K.A. Spice Phenolics Inhibit Human PMNL 5-Lipoxygenase. *Prostaglandins Leukot. Essent. Fatty Acids* **2004**, *70*, 521–528, doi:10.1016/j.plefa.2003.11.006.
43. Hsu, M.-F.; Lu, M.-C.; Tsao, L.-T.; Kuan, Y.-H.; Chen, C.-C.; Wang, J.-P. Mechanisms of the Influence of Magnolol on Eicosanoid Metabolism in Neutrophils. *Biochem. Pharmacol.* **2004**, *67*, 831–840, doi:10.1016/j.bcp.2003.09.040.
44. Son, J.K.; Son, M.J.; Lee, E.; Moon, T.C.; Son, K.H.; Kim, C.-H.; Kim, H.P.; Kang, S.S.; Chang, H.W. Ginkgetin, a Biflavone from Ginko Biloba Leaves, Inhibits Cyclooxygenases-2 and 5-Lipoxygenase in Mouse Bone Marrow-Derived Mast Cells. *Biol. Pharm. Bull.* **2005**, *28*, 2181–2184, doi:10.1248/bpb.28.2181.
45. Bas, E.; Recio, M.C.; Giner, R.M.; Máñez, S.; Cerdá-Nicolás, M.; Ríos, J.-L. Anti-Inflammatory Activity of 5- *O* -Demethylnobiletin, a Polymethoxyflavone Isolated from *Sideritis Tragoriganum*. *Planta Med.* **2006**, *72*, 136–142, doi:10.1055/s-2005-873191.
46. Chen, D.-M.; Cai, X.; Kwik-Uribe, C.L.; Zeng, R.; Zhu, X.-Z. Inhibitory Effects of Procyanidin B(2) Dimer on Lipid-Laden Macrophage Formation. *J. Cardiovasc. Pharmacol.* **2006**, *48*, 54–70, doi:10.1097/01.fjc.0000242052.60502.21.
47. Kim, J.S.; Kim, J.C.; Shim, S.H.; Lee, E.J.; Jin, W.; Bae, K.; Son, K.H.; Kim, H.P.; Kang, S.S.; Chang, H.W. Chemical Constituents of the Root of Dystaenia Takeshimana and Their Anti-Inflammatory Activity. *Arch. Pharm. Res.* **2006**, *29*, 617–623, doi:10.1007/BF02968244.
48. Kim, S.J.; Jin, M.; Lee, E.; Moon, T.C.; Quan, Z.; Yang, J.H.; Son, K.H.; Kim, K.-U.; Son, J.K.; Chang, H.W. Effects of Methyl Gallate on Arachidonic Acid Metabolizing Enzymes: Cyclooxygenase-2 and 5-Lipoxygenase in Mouse Bone Marrow-Derived Mast Cells. *Arch. Pharm. Res.* **2006**, *29*, 874–878, doi:10.1007/BF02973908.
49. Son, M.J.; Moon, T.C.; Lee, E.K.; Son, K.H.; Kim, H.P.; Kang, S.S.; Son, J.K.; Lee, S.H.; Chang, H.W. Naturally Occurring Biflavonoid, Ochnaflavone, Inhibits Cyclooxygenases-2 and 5-Lipoxygenase in Mouse Bone Marrow-Derived Mast Cells. *Arch. Pharm. Res.* **2006**, *29*, 282–286, doi:10.1007/BF02968571.
50. Raghavenra, H.; Diwakr, B.T.; Lokesh, B.R.; Naidu, K.A. Eugenol--the Active Principle from Cloves Inhibits 5-Lipoxygenase Activity and Leukotriene-C4 in Human PMNL Cells. *Prostaglandins Leukot. Essent. Fatty Acids* **2006**, *74*, 23–27, doi:10.1016/j.plefa.2005.08.006.
51. Kalhan, R.; Smith, L.J.; Nlend, M.C.; Nair, A.; Hixon, J.L.; Sporn, P.H.S. A Mechanism of Benefit of Soy Genistein in Asthma: Inhibition of Eosinophil P38-Dependent Leukotriene Synthesis. *Clin. Exp. Allergy* **2007**, *0*, 071104193151002-???, doi:10.1111/j.1365-2222.2007.02862.x.
52. Hernández, V.; Recio, M.C.; Máñez, S.; Giner, R.M.; Ríos, J.-L. Effects of Naturally Occurring Dihydroflavonols from Inula Viscosa on Inflammation and Enzymes Involved in the Arachidonic Acid Metabolism. *Life Sci.* **2007**, *81*, 480–488, doi:10.1016/j.lfs.2007.06.006.

53. Park, S.; Han, S.-U.; Lee, K.-M.; Park, K.H.; Cho, S.W.; Hahm, K.-B. 5-LOX Inhibitor Modulates the Inflammatory Responses Provoked by Helicobacter Pylori Infection. *Helicobacter* **2007**, *12*, 49–58, doi:10.1111/j.1523-5378.2007.00469.x.
54. Moon, T.C.; Seo, C.S.; Haa, K.; Kim, J.C.; Hwang, N.K.; Hong, T.G.; Kim, J.H.; Kim, D.H.; Son, J.K.; Chang, H.W. Meso-Dihydroguaiaretic Acid Isolated from Saururus Chinensis Inhibits Cyclooxygenase-2 and 5-Lipoxygenase in Mouse Bone Marrow-Derived Mast Cells. *Arch. Pharm. Res.* **2008**, *31*, 606–610, doi:10.1007/s12272-001-1200-y.
55. Poeckel, D.; Greiner, C.; Verhoff, M.; Rau, O.; Tausch, L.; Hörnig, C.; Steinhilber, D.; Schubert-Zsilavecz, M.; Werz, O. Carnosic Acid and Carnosol Potently Inhibit Human 5-Lipoxygenase and Suppress pro-Inflammatory Responses of Stimulated Human Polymorphonuclear Leukocytes. *Biochem. Pharmacol.* **2008**, *76*, 91–97, doi:10.1016/j.bcp.2008.04.013.
56. Loke, W.M.; Proudfoot, J.M.; Stewart, S.; McKinley, A.J.; Needs, P.W.; Kroon, P.A.; Hodgson, J.M.; Croft, K.D. Metabolic Transformation Has a Profound Effect on Anti-Inflammatory Activity of Flavonoids Such as Quercetin: Lack of Association between Antioxidant and Lipoxygenase Inhibitory Activity. *Biochem. Pharmacol.* **2008**, *75*, 1045–1053, doi:10.1016/j.bcp.2007.11.002.
57. Anogianaki, A.; Castellani, M.L.; Madhappan, B.; Salini, V.; Vecchiet, J.; Tetè, S.; Frydas, S.; Perrella, A.; De Lutiis, M.A.; Neri, G.; et al. RANTES (CCL5) Potentiates Calcium Ionophore in the Production of LTB4 in Rat Adherent Macrophages from Granuloma Induced by KMnO4: Inhibiton by NDGA. *Pharmacol. Res.* **2008**, *57*, 49–55, doi:10.1016/j.phrs.2007.11.002.
58. Castellani, M.L.; Conti, P.; Felaco, M.; Vecchiet, J.; Ciampoli, C.; Cerulli, G.; Boscolo, P.; Theoharides, T.C. Substance P Upregulates LTB4 in Rat Adherent Macrophages from Granuloma Induced by KMnO4. *Neurotox. Res.* **2009**, *15*, 49–56, doi:10.1007/s12640-009-9004-6.
59. Jin, M.-I.; Bae, K.-H.; Chang, H.-W.; Son, J.-K. Anti-Inflammatory Compounds from the Leaves of Ailanthus Altissima Meihua JIN. *Biomol. Ther.* **2009**, *17*, 86–91, doi:10.4062/biomolther.2009.17.1.86.
60. Feisst, C.; Pergola, C.; Rakonjac, M.; Rossi, A.; Koeberle, A.; Dodt, G.; Hoffmann, M.; Hoernig, C.; Fischer, L.; Steinhilber, D.; et al. Hyperforin Is a Novel Type of 5-Lipoxygenase Inhibitor with High Efficacy in Vivo. *Cell. Mol. Life Sci. CMLS* **2009**, *66*, 2759–2771, doi:10.1007/s00018-009-0078-3.
61. Lim, H.; Nam, J.W.; Seo, E.-K.; Kim, Y.S.; Kim, H.P. (-)-Nyasol (Cis-Hinokiresinol), a Norneolignan from the Rhizomes of Anemarrhena Asphodeloides, Is a Broad Spectrum Inhibitor of Eicosanoid and Nitric Oxide Production. *Arch. Pharm. Res.* **2009**, *32*, 1509–1514, doi:10.1007/s12272-009-2102-4.
62. Lim, H.; Son, K.H.; Bae, K.H.; Hung, T.M.; Kim, Y.S.; Kim, H.P. 5-Lipoxygenase-Inhibitory Constituents from Schizandra Fructus and Magnolia Flos. *Phytother. Res. PTR* **2009**, *23*, 1489–1492, doi:10.1002/ptr.2783.
63. Kim, J.M.; Lee, E.K.; Park, G.; Kim, M.K.; Yokozawa, T.; Yu, B.P.; Chung, H.Y. Morin Modulates the Oxidative Stress-Induced NF-KappaB Pathway through Its Anti-Oxidant Activity. *Free Radic. Res.* **2010**, *44*, 454–461, doi:10.3109/10715761003610737.
64. Saw, C.L.L.; Huang, Y.; Kong, A.-N. Synergistic Anti-Inflammatory Effects of Low Doses of Curcumin in Combination with Polyunsaturated Fatty Acids: Docosahexaenoic Acid or Eicosapentaenoic Acid. *Biochem. Pharmacol.* **2010**, *79*, 421–430, doi:10.1016/j.bcp.2009.08.030.

65. Lin, T.-Y.; London, C.A. Characterization and Modulation of Canine Mast Cell Derived Eicosanoids. *Vet. Immunol. Immunopathol.* **2010**, *135*, 118–127, doi:10.1016/j.vetimm.2009.11.010.
66. Lee, E.J.; Kim, J.S.; Kim, H.P.; Lee, J.-H.; Kang, S.S. Phenolic Constituents from the Flower Buds of Lonicera Japonica and Their 5-Lipoxygenase Inhibitory Activities. *Food Chem.* **2010**, *120*, 134–139, doi:10.1016/j.foodchem.2009.09.088.
67. Bhaskar, S.; Shalini, V.; Helen, A. Quercetin Regulates Oxidized LDL Induced Inflammatory Changes in Human PBMCs by Modulating the TLR-NF-KB Signaling Pathway. *Immunobiology* **2011**, *216*, 367–373, doi:10.1016/j.imbio.2010.07.011.
68. Kwon, O.S.; Choi, J.S.; Islam, Md.N.; Kim, Y.S.; Kim, H.P. Inhibition of 5-Lipoxygenase and Skin Inflammation by the Aerial Parts of Artemisia Capillaris and Its Constituents. *Arch. Pharm. Res.* **2011**, *34*, 1561–1569, doi:10.1007/s12272-011-0919-0.
69. Fan, S.-Y.; Zeng, H.-W.; Pei, Y.-H.; Li, L.; Ye, J.; Pan, Y.-X.; Zhang, J.-G.; Yuan, X.; Zhang, W.-D. The Anti-Inflammatory Activities of an Extract and Compounds Isolated from Platycladus Orientalis (Linnaeus) Franco in Vitro and Ex Vivo. *J. Ethnopharmacol.* **2012**, *141*, 647–652, doi:10.1016/j.jep.2011.05.019.
70. Lee, J.H.; Lee, D.U.; Kim, Y.S.; Kim, H.P. 5-Lipoxygenase Inhibition of the Fructus of Foeniculum Vulgare and Its Constituents. *Biomol. Ther.* **2012**, *20*, 113–117, doi:10.4062/biomolther.2012.20.1.113.
71. Han, Y.S.; Kim, M.-S.; Hwang, J.-K. Macelignan Inhibits Histamine Release and Inflammatory Mediator Production in Activated Rat Basophilic Leukemia Mast Cells. *Inflammation* **2012**, *35*, 1723–1731, doi:10.1007/s10753-012-9490-1.
72. Boudreau, L.H.; Maillet, J.; LeBlanc, L.M.; Jean-François, J.; Touaibia, M.; Flamand, N.; Surette, M.E. Caffeic Acid Phenethyl Ester and Its Amide Analogue Are Potent Inhibitors of Leukotriene Biosynthesis in Human Polymorphonuclear Leukocytes. *PloS One* **2012**, *7*, e31833, doi:10.1371/journal.pone.0031833.
73. Choi, Y.; Kim, M.S.; Hwang, J.-K. Inhibitory Effects of Panduratin A on Allergy-Related Mediator Production in Rat Basophilic Leukemia Mast Cells. *Inflammation* **2012**, *35*, 1904–1915, doi:10.1007/s10753-012-9513-y.
74. Kogut, M.H.; He, H.; Genovese, K.J. Bacterial Toll-like Receptor Agonists Induce Sequential NF-KB-Mediated Leukotriene B4 and Prostaglandin E2 Production in Chicken Heterophils. *Vet. Immunol. Immunopathol.* **2012**, *145*, 159–170, doi:10.1016/j.vetimm.2011.11.003.
75. Lim, J.C.; Park, S.Y.; Nam, Y.; Nguyen, T.T.; Sohn, U.D. The Protective Effect of Eupatilin against Hydrogen Peroxide-Induced Injury Involving 5-Lipoxygenase in Feline Esophageal Epithelial Cells. *Korean J. Physiol. Pharmacol. Off. J. Korean Physiol. Soc. Korean Soc. Pharmacol.* **2012**, *16*, 313–320, doi:10.4196/kjpp.2012.16.5.313.
76. Gomes, A.; Couto, D.; Alves, A.; Dias, I.; Freitas, M.; Porto, G.; Duarte, J.A.; Fernandes, E. Trihydroxyflavones with Antioxidant and Anti-Inflammatory Efficacy. *BioFactors Oxf. Engl.* **2012**, *38*, 378–386, doi:10.1002/biof.1033.
77. Li, L.; Zeng, H.; Shan, L.; Yuan, X.; Li, Y.; Liu, R.; Zhang, W. The Different Inhibitory Effects of Huang-Lian-Jie-Du-Tang on Cyclooxygenase 2 and 5-Lipoxygenase. *J. Ethnopharmacol.* **2012**, *143*, 732–739, doi:10.1016/j.jep.2012.07.037.

78. Werz, O.; Seegers, J.; Schaible, A.M.; Weinigel, C.; Barz, D.; Koeberle, A.; Allegrone, G.; Pollastro, F.; Zampieri, L.; Grassi, G.; et al. Cannflavins from Hemp Sprouts, a Novel Cannabinoid-Free Hemp Food Product, Target Microsomal Prostaglandin E2 Synthase-1 and 5-Lipoxygenase. *PharmaNutrition* **2014**, *2*, 53–60, doi:10.1016/j.phanu.2014.05.001.
79. Ribeiro, D.; Freitas, M.; Tomé, S.M.; Silva, A.M.S.; Porto, G.; Cabrita, E.J.; Marques, M.M.B.; Fernandes, E. Inhibition of LOX by Flavonoids: A Structure-Activity Relationship Study. *Eur. J. Med. Chem.* **2014**, *72*, 137–145, doi:10.1016/j.ejmech.2013.11.030.
80. Lee, Y.S.; Hur, S.; Kim, T.-Y. Homoisoflavanone Prevents Mast Cell Activation and Allergic Responses by Inhibition of Syk Signaling Pathway. *Allergy* **2014**, *69*, 453–462, doi:10.1111/all.12356.
81. Li, X.; Lu, Y.; Jin, Y.; Son, J.-K.; Lee, S.H.; Chang, H.W. Curcumin Inhibits the Activation of Immunoglobulin E-Mediated Mast Cells and Passive Systemic Anaphylaxis in Mice by Reducing Serum Eicosanoid and Histamine Levels. *Biomol. Ther.* **2014**, *22*, 27–34, doi:10.4062/biomolther.2013.092.
82. Kutil, Z.; Kvasnicova, M.; Temml, V.; Schuster, D.; Marsik, P.; Cusimamani, E.F.; Lou, J.-D.; Vanek, T.; Landa, P. Effect of Dietary Stilbenes on 5-Lipoxygenase and Cyclooxygenases Activities In Vitro. *Int. J. Food Prop.* **2015**, *18*, 1471–1477, doi:10.1080/10942912.2014.903416.
83. Forino, M.; Pace, S.; Chianese, G.; Santagostini, L.; Werner, M.; Weinigel, C.; Rummler, S.; Fico, G.; Werz, O.; Taglialatela-Scafati, O. Humudifucol and Bioactive Prenylated Polyphenols from Hops (Humulus Lupulus Cv. "Cascade"). *J. Nat. Prod.* **2016**, *79*, 590–597, doi:10.1021/acs.jnatprod.5b01052.
84. Tsen, S.Y.; Tan, X.Y.; Tan, Y.M.; Yan, B.Y.; Loke, W.M. Relative Inhibitions of 5-Lipoxygenase and Myeloperoxidase and Free-Radical Scavenging Activities of Daidzein, Dihydrodaidzein, and Equol. *J. Med. Food* **2016**, *19*, 543–548, doi:10.1089/jmf.2015.3557.
85. Roy, P.-P.; Faye, D.; Blanchard, S.; Cormier, M.; Doiron, J.A.; Surette, M.E.; Touaibia, M. New Caffeic Acid Phenylethyl Ester Analogs Bearing Substituted Triazole: Synthesis and Structure-Activity Relationship Study towards 5-Lipoxygenase Inhibition. *J. Chem.* **2017**, *2017*, e2380531, doi:10.1155/2017/2380531.
86. Anilkumar, K.; Reddy, G.V.; Azad, R.; Yarla, N.S.; Dharmapuri, G.; Srivastava, A.; Kamal, M.A.; Pallu, R. Evaluation of Anti-Inflammatory Properties of Isoorientin Isolated from Tubers of *Pueraria Tuberosa*. *Oxid. Med. Cell. Longev.* **2017**, *2017*, 1–10, doi:10.1155/2017/5498054.
87. Takasugi, M.; Muta, E.; Yamada, K.; Arai, H. A New Method to Evaluate Anti-Allergic Effect of Food Component by Measuring Leukotriene B4 from a Mouse Mast Cell Line. *Cytotechnology* **2018**, *70*, 177–184, doi:10.1007/s10616-017-0129-9.
88. Doucet, M.S.; Jougleux, J.-L.; Poirier, S.J.; Cormier, M.; Léger, J.L.; Surette, M.E.; Pichaud, N.; Touaibia, M.; Boudreau, L.H. Identification of Peracetylated Quercetin as a Selective 12-Lipoxygenase Pathway Inhibitor in Human Platelets. *Mol. Pharmacol.* **2019**, *95*, 139–150, doi:10.1124/mol.118.113480.
89. Mbarik, M.; Poirier, S.J.; Doiron, J.; Selka, A.; Barnett, D.A.; Cormier, M.; Touaibia, M.; Surette, M.E. Phenolic Acid Phenethylesters and Their Corresponding Ketones: Inhibition of 5-Lipoxygenase and Stability in Human Blood and HepaRG Cells. *Pharmacol. Res. Perspect.* **2019**, *7*, e00524, doi:10.1002/prp2.524.
90. Storniolo, C.E.; Martínez-Hovelman, N.; Martínez-Huélamo, M.; Lamuela-Raventos, R.M.; Moreno, J.J. Extra Virgin Olive Oil Minor Compounds Modulate Mitogenic Action of Oleic Acid on Colon Cancer Cell Line. *J. Agric. Food Chem.* **2019**, *67*, 11420–11427, doi:10.1021/acs.jafc.9b04816.

91. Prabha, B.; Sini, S.; Priyadarshini, T.S.; Sasikumar, P.; Gopalan, G.; Joseph, J.P.; Jithin, M.M.; Sivan, V.V.; Jayamurthy, P.; Radhakrishnan, K.V. Anti-Inflammatory Effect and Mechanism of Action of Ellagic Acid-3,3′,4-Trimethoxy-4′-O-α-L-Rhamnopyranoside Isolated from Hopea Parviflora in Lipopolysaccharide-Stimulated RAW 264.7 Macrophages. *Nat. Prod. Res.* **2019**, 1–5, doi:10.1080/14786419.2019.1690486.
92. Gerstmeier, J.; Seegers, J.; Witt, F.; Waltenberger, B.; Temml, V.; Rollinger, J.M.; Stuppner, H.; Koeberle, A.; Schuster, D.; Werz, O. Ginkgolic Acid Is a Multi-Target Inhibitor of Key Enzymes in Pro-Inflammatory Lipid Mediator Biosynthesis. *Front. Pharmacol.* **2019**, *10*, 797, doi:10.3389/fphar.2019.00797.
93. Hartung, N.M.; Fischer, J.; Ostermann, A.I.; Willenberg, I.; Rund, K.M.; Schebb, N.H.; Garscha, U. Impact of Food Polyphenols on Oxylipin Biosynthesis in Human Neutrophils. *Biochim. Biophys. Acta Mol. Cell Biol. Lipids* **2019**, *1864*, 1536–1544, doi:10.1016/j.bbalip.2019.05.002.
94. Jin, S.E.; Ha, H.; Shin, H.-K.; Seo, C.-S. Anti-Allergic and Anti-Inflammatory Effects of Kuwanon G and Morusin on MC/9 Mast Cells and HaCaT Keratinocytes. *Mol. Basel Switz.* **2019**, *24*, E265, doi:10.3390/molecules24020265.
95. Pan, S.; Zhao, X.; Ji, N.; Shao, C.; Fu, B.; Zhang, Z.; Wang, R.; Qiu, Y.; Jin, M.; Kong, D. Inhibitory Effect of Taxifolin on Mast Cell Activation and Mast Cell-Mediated Allergic Inflammatory Response. *Int. Immunopharmacol.* **2019**, *71*, 205–214, doi:10.1016/j.intimp.2019.03.038.
96. Giménez-Bastida, J.A.; González-Sarrías, A.; Espín, J.C.; Schneider, C. Inhibition of 5-Lipoxygenase-Derived Leukotrienes and Hemiketals as a Novel Anti-Inflammatory Mechanism of Urolithins. *Mol. Nutr. Food Res.* **2020**, *64*, e2000129, doi:10.1002/mnfr.202000129.
97. Habib, E.S.; El-Bsoumy, E.; Ibrahim, A.K.; Helal, M.A.; El-Magd, M.A.; Ahmed, S.A. Anti-Inflammatory Effect of Methoxyflavonoids from *Chiliadenus Montanus* (*Jasonia Montana)* Growing in Egypt. *Nat. Prod. Res.* **2020**, 1–5, doi:10.1080/14786419.2020.1802272.
98. Gilbert, N.C.; Gerstmeier, J.; Schexnaydre, E.E.; Börner, F.; Garscha, U.; Neau, D.B.; Werz, O.; Newcomer, M.E. Structural and Mechanistic Insights into 5-Lipoxygenase Inhibition by Natural Products. *Nat. Chem. Biol.* **2020**, *16*, 783–790, doi:10.1038/s41589-020-0544-7.
99. Mostafa, E.S.; Nawwar, M.A.M.; Mostafa, D.A.; Ragab, M.F.; Swilam, N. Karafsin, a Unique Mono-Acylated Flavonoid Apiofurnoside from the Leaves of Apium Graveolens Var. Secalinum Alef: In Vitro and in Vivo Anti-Inflammatory Assessment. *Ind. Crops Prod.* **2020**, *158*, 112901, doi:10.1016/j.indcrop.2020.112901.
100. Lee, J.-Y.; Park, S.-H.; Jhee, K.-H.; Yang, S.-A. Tricin Isolated from Enzyme-Treated Zizania Latifolia Extract Inhibits IgE-Mediated Allergic Reactions in RBL-2H3 Cells by Targeting the Lyn/Syk Pathway. *Mol. Basel Switz.* **2020**, *25*, E2084, doi:10.3390/molecules25092084.
101. Toda, K.; Tsukayama, I.; Nagasaki, Y.; Konoike, Y.; Tamenobu, A.; Ganeko, N.; Ito, H.; Kawakami, Y.; Takahashi, Y.; Miki, Y.; et al. Red-Kerneled Rice Proanthocyanidin Inhibits Arachidonate 5-Lipoxygenase and Decreases Psoriasis-like Skin Inflammation. *Arch. Biochem. Biophys.* **2020**, *689*, 108307, doi:10.1016/j.abb.2020.108307.
